# Evolution of an epidemic: Understanding the opioid epidemic in the United States and the impact of the COVID-19 pandemic on opioid-related mortality

**DOI:** 10.1371/journal.pone.0306395

**Published:** 2024-07-09

**Authors:** Rachel Laing, Christl A. Donnelly

**Affiliations:** 1 Department of Statistics, University of Oxford, Oxford, United Kingdom; 2 Division of Infectious Diseases, Massachusetts General Hospital, Cambridge, Massachusetts, United States of America; 3 Pandemic Sciences Institute, University of Oxford, Oxford, United Kingdom; 4 MRC Centre for Global Infectious Disease Analysis, Imperial College London, London, United Kingdom; University of Toronto, CANADA

## Abstract

We conduct this research with a two-fold aim: providing a quantitative analysis of the opioid epidemic in the United States (U.S.), and exploring the impact of the COVID-19 pandemic on opioid-related mortality. The duration and persistence of the opioid epidemic lends itself to the need for an overarching analysis with extensive scope. Additionally, studying the ramifications of these concurrent severe public health crises is vital for informing policies to avoid preventable mortality. Using data from CDC WONDER, we consider opioid-related deaths grouped by Census Region spanning January 1999 to October 2022 inclusive, and later add on a demographic component with gender-stratification. Through the lens of key events in the opioid epidemic, we build an interrupted time series model to reveal statistically significant drivers of opioid-related mortality. We then employ a counterfactual to approximate trends in the absence of COVID-19, and estimate excess opioid-related deaths (defined as observed opioid-related deaths minus projected opioid-related deaths) associated with the pandemic. According to our model, the proliferation of fentanyl contributed to sustained increases in opioid-related death rates across three of the four U.S. census regions, corroborating existing knowledge in the field. Critically, each region has an immediate increase to its opioid-related monthly death rate of at least 0.31 deaths per 100,000 persons at the start of the pandemic, highlighting the nationwide knock-on effects of COVID-19. There are consistent positive deviations from the expected monthly opioid-related death rate and a sizable burden from cumulative excess opioid-related deaths, surpassing 60,000 additional deaths nationally from March 2020 to October 2022, ∼70% of which were male. These results suggest that robust, multi-faceted measures are even more important in light of the COVID-19 pandemic to prevent overdoses and educate users on the risks associated with potent synthetic opioids such as fentanyl.

## Introduction

The United States (U.S.) has been battling an opioid epidemic since the 1990s [1]. Typically characterized by three waves, overdose deaths from prescription opioids started rising in 1996 due to increased prescription rates and the introduction of OxyContin [2]. Heroin contributed to a second wave in 2010 which was especially severe in the Northeast and South census regions (CRs) [3]. The prevalence of synthetic opioids, primarily illicit fentanyl, drove a sharp third wave commencing in 2013 [4]. By the time the crisis was declared a public health emergency (PHE) on October 26, 2017 [5], opioid overdoses had already claimed hundreds of thousands of lives. The onset of the COVID-19 pandemic in early 2020 resulted in concurrent health crises, amplifying the burden of opioid mortality and arguably catalyzing a fourth wave of the opioid epidemic [6]. For ease of notation, we may hereafter refer to the opioid epidemic as the “epidemic” and the COVID-19 pandemic as the “pandemic”.

### Opioid usage

Opioid use disorder (OUD) is a chronic psychiatric condition characterized by “a desire to obtain and take opioids despite social and professional consequences” [7]. It is well-established that widespread availability of prescription opioids contributed to a long-running pattern of fatalities, as their addictive nature would lead to misuse and OUDs [8]. Individuals with addictions would then often seek out heroin and other illicit forms of opioids, which are easier and cheaper to obtain [9]. The death toll due to opioid mortality is largely comprised of individuals with OUDs, who are at the highest risk of overdose [10]. To address OUDs, medication-assisted treatment (MAT) is often employed in conjunction with psychotherapy in severe cases with the aim of remission [11, 12].

In the realm of recreational usage, illicit fentanyl and its analogues introduced an additional element of lethality due to their rapid effect time and high potency compared to other opioids, such as oxycodone and heroin [13]. Less potent drugs (e.g. heroin, cocaine, methamphetamine) laced with synthetic fentanyl are also a widespread concern linked with accidental overdose deaths [14, 15].

### COVID-19 impact

Existing literature on the intersection between COVID-19 and the opioid epidemic outlines the challenges impacting individuals vulnerable to opioid misuse and abuse.

The COVID-19 pandemic drastically impacted psychiatric healthcare provisions. With the rise of telehealth, lack of in-person consultations led to patient apprehension regarding MAT. Fear of visiting hospitals for laboratory work and specialist-administered medication due to the risk of SARS-CoV-2 transmission also created barriers in the treatment process [16]. Early articles address the role of opioid treatment clinic closures and emergency department prioritization of COVID-19 patients in creating treatment access barriers for OUD patients [17–20]. In June 2020, researchers theorized a fourth wave of the opioid epidemic, citing the trade-off in substance detoxification and inpatient programs for pandemic relief. Prescriptions of self-administered MAT became commonplace, but involve dangers of accidental misuse in isolation [6]. Disrupted support systems and delayed treatment may drive patients to self-manage with illicit opioids [21]. Individuals with undiagnosed or untreated OUDs are especially vulnerable, and face higher mortality rates than those who receive treatment [22]. Additionally, any overdoses occurring in isolation without naloxone access due to social distancing are likely to be fatal [20, 23].

The mental health impacts due to the pandemic have also been profound. Psychological distress including, but not limited to, stress, anxiety, depression, and loneliness became more prevalent as a result of social distancing, fear regarding the SARS-CoV-2 pathogen, and the unprecedented nature of a global pandemic [24–28]. These ramifications were even more severe for groups with existing psychiatric vulnerabilities, leading to an increased incidence of anxiety and depression symptoms and harmful substance usage [29–33]. Evidence suggests that mental health disorders and existing drug misuse are each associated with higher rates of opioid use [34, 35], and thus are also root causes for opioid-related deaths [36]. There are also high rates of comorbidity between mood disorders and substance use disorders generally [37]. Hence, it is reasonable to postulate a link between the pandemic and increased opioid usage, overdoses, and opioid-related deaths via transitivity.

These crises have disproportionately impacted low-income and homeless OUD patients who face increased risks of contracting COVID-19 whilst unable to socially distance, but are also more likely to misuse drugs due to pandemic distress [18]. Adult men have been particularly impacted by deaths of despair, including via opioid overdoses, during the onset of the pandemic [38]. Social marginalization and infectious disease comorbidities further increase the risk of fatal opioid overdoses [39, 40]. Shortcomings in opioid treatment protocols warrant alternatives that are robust against social restrictions, such as resiliency building and stress management [41].

The six-month lag in overdose data reporting [42] made it impossible to examine real-time COVID-19 impacts on the opioid epidemic. Retrospective data-driven approaches started appearing in September 2020. Through descriptive statistics, a Virginia emergency department found that nonfatal overdose visits increased early in the pandemic whilst follow-up consultation rates were low [43]. A study of patient visits at a hospital system in Pennsylvania before and after a shelter-in-place (SIP) order reported an overall decrease in counts of both general and opioid-related visits, but a statistically significant increase in the proportion of opioid overdoses [44]. Another paper proposed the merits of linked patient data to evaluate individual behavioral changes during the pandemic [45]. Whilst drug testing declined, urine specimen analysis uncovered increases in positivity rates for illicit fentanyl and heroin [46]. Significant increases in illicit fentanyl positivity rates were found in specimens positive for substances such as heroin, cocaine, and amphetamines, suggesting a rise in lethal drug combinations [46]. From a geographical viewpoint with county-level granularity, significant positive associations were discovered between COVID-19 mortality and opioid-related mortality [47]. A joinpoint analysis covering 11 states demonstrated statistically significant changes in annual opioid overdose deaths for 7 states between 2019 and 2020, compared to only 1 state between 2018 and 2019 [48].

The literature also contains efforts to quantify the burden of the opioid epidemic in the wake of COVID-19. One study counted over 68,000 opioid overdose deaths in 2020, and found that trends in opioid-related death rates during the first year of the pandemic were independent of Medicaid expansion status [49], but without comparing these death tolls to baseline predictions, it is impossible to contextualize how many of those deaths were attributable to the pandemic. Tangential excess death estimates include ∼30,000 non-COVID-19 excess deaths nationally from March 2020 to October 2020 [38] and ∼2,000 excess fatal drug overdoses in California from January 2020 to December 2020 [50], both of which would include excess opioid-related deaths as a subset. An overall increase in cumulative excess mortality was also shown in Massachusetts from March 2020 to March 2021 [51]. However, to the best of our knowledge at the time of writing, there is no existing research quantifying excess opioid-related deaths due to COVID-19 on a national level in the U.S.

### Aim

This research aims to provide a detailed analysis of the opioid epidemic in the U.S., with a particular focus on the sensitivity of opioid-related mortality to a large-scale concurrent disaster, namely the COVID-19 pandemic. We endeavor to provide a unique contribution to the literature through our multi-faceted approach in modelling the opioid epidemic. Understanding the trajectory of the opioid epidemic through key events is vital to informing public health initiatives and responses, if there is to be substantial progress in mitigating this source of preventable deaths. We purport that insight can be gained via an approach founded in regression modeling, which allows for results to be easily interpretable and reproducible.

After briefly describing the data, processing, and methodology, we formulate a novel interrupted time series (ITS) model based on a high-level overview of the opioid epidemic which can be easily modified with interaction terms to capture demographic characteristics of interest. We also explain how to calculate excess and cumulative excess opioid-related deaths due to COVID-19 using the counterfactual. We then explore the model output, diagnostics, and accompanying figures in order to quantify opioid epidemic trends in different CRs, and conclude with a discussion on how these results can aid in future harm-prevention efforts.

## Methods

In this section, we detail the methodology and mathematical foundations used for each step from data collection, modeling, and analysis to establish a robust framework for high-level epidemic trajectory analysis.

### Data

We use Multiple Cause of Death (MCOD) data from the Wide-Ranging Online Data for Epidemiological Research (WONDER) online database provided by the Centers for Disease Control and Prevention (CDC) [52, 53]. The CDC WONDER database system allows for users to make queries and download the resulting tables for analysis [54, 55]. The MCOD data itself is drawn from death certificates of U.S. residents, which contain underlying cause(s) of death along with demographic data [53]. MCOD data has been utilized for a variety of public health and mortality research endeavors [56–61], including in the context of opioid-related deaths [62–64]. Drug-related mortality analysis faces limitations due to death certificate reporting quality and state-level variation in medical examiner systems, leading to undercounting [65, 66]. However, since reported statistics are an underestimate, our model results are thus conservative; true increases in opioid-related death rates and excess death counts may be even more severe than the analysis predicts.

The MCOD data is available in two forms: current final data from 1999 to 2020, and provisional data from 2018 up to the previous month at the time of request. Typically the most recent four to six months of data are classed as partial due to delays in death reporting [42]. Hence, in correspondence with our dataset download on May 17, 2023, the scope of analysis ends conservatively in October 2022. We stratify our opioid data by CR and gender, which allows us to characterize demographic variation over the course of the opioid epidemic. Note that CR indicates the residence area associated with the individual who died, which may not necessarily be the region in which the death occurred. Additional information on CRs can be found in S1 Appendix. Since data request entries with counts below ten are suppressed by the CDC, we work with monthly data to avoid granularity-induced limitations. This also happens to be the shortest time interval available to us across the entire scope of analysis, as weekly data is only available from 2018 onwards at a provisional status. We then merge final and provisional data to create a dataset spanning January 1999 to October 2022, which ensures we have recent data to quantify the impacts of the pandemic as well as sufficient historic data for predictions and trends over time. By incorporating the early data into our analysis, we gain confidence that we can model trends in the absence of COVID-19, which therefore gives us more confidence that the resulting predictions are valid estimations of what would have happened.

The full working dataset contains observations at the CR—Gender—Year—Month level, so we simply aggregate over the gender variable in order to examine CRs as a whole.

#### ICD-10 codes for opioid-related deaths

The International Classification of Diseases (ICD) is a set of codes published by the World Health Organization (WHO) which provides a standardized system for analyzing mortality and morbidity [67]. Whilst the ICD’s 11^th^ revision is now in effect as of January 2022 [68], this work relies on ICD-10 codes as per CDC WONDER entries.

In order to restrict to opioid-related deaths, we first utilize the following drug-related **X** and **Y** ICD-10 codes for underlying cause of death:

- **X40–44**: accidental poisoning;
- **X60–64**: intentional self-poisoning;
- **X85**: assault through substances;
- **Y10–14**: poisoning with undetermined intent.

These are then paired with **T** codes identifying contributing cause of death. This allows us to pick out drug poisonings related to the opioids of choice listed below:

- **T40.0**: poisoning by opium;
- **T40.1**: poisoning by heroin;
- **T40.2**: poisoning by other opioids;
- **T40.3**: poisoning by methadone;
- **T40.4**: poisoning by synthetic narcotics;
- **T40.6**: poisoning by other and unspecified narcotics.

In this research, we group all of these codes together for an overarching view of the epidemic.

### Date approximations

The MCOD data provides observations at a monthly granularity, but we need to convert these to date formats in order to utilize the time series structure. As the death count for each observation encompasses all deaths that occur over the course of that month, we assign each value to the final day of its respective month (e.g. January deaths correspond to January 31, April deaths correspond to April 30, etc).

### Population interpolation

Since we stratify analysis by CR and/or gender, death rates $\left(\frac{\text{deaths}}{\text{population}}\right)$ are essential for making meaningful comparisons across groups with varying subpopulation sizes. The CDC WONDER database only provides population estimates on a yearly basis, so we must estimate monthly population sizes in order to obtain monthly death rates. The population data was downloaded on May 17, 2023, and includes values for 2023 though they are identical to those for 2022. For simplicity and based on MCOD conventions regarding U.S. Census Bureau data, we assign the annual population estimates to July 1st of the year. We then use Python’s built-in ‘time’ interpolation method, which is essentially linear interpolation for datasets a datetime index, to obtain population size estimates for the dates corresponding to death counts between July 1999 and October 2022, and ‘backfill’ interpolation for January 1999 to June 1999 [69].

### Death rates

Given monthly opioid-related death count *d*_*t*,*g*_ and monthly interpolated population estimate *p*_*t*,*g*_ for subpopulation group *g* at time *t*, the corresponding monthly opioid-related death rate is

$$\begin{array}{c} y_{t,g}=100000\times (\frac{d_{t,g}}{p_{t,g}}), \end{array}$$ (1)

which represents the number of opioid-related deaths per 100,000 persons. Hereafter, we forgo the subscript *g* in notation, but it remains that death rates are always relative to the subpopulation of interest.

### Linear regression

Linear regression models an outcome variable *y* as a linear combination of *p* explanatory variables [70]. This is expressed as

$$\begin{array}{c} y=\beta_{0}+\beta_{1}x_{1}+\beta_{2}x_{2}+\cdots +\beta_{p}x_{p}+\epsilon , \end{array}$$ (2)

where the *β*_*i*_’s denote regression coefficients, *x*_*i*_’s are explanatory variables, and *ϵ* is an error term. The four critical assumptions are:

- linear relationship between outcome and explanatory variables;
- independent observations;
- normality of error terms;
- homoscedasticity, or equal variance, of error terms [71].

### Interrupted time series

Interrupted time series (ITS) analysis is useful for evaluating the effect of an intervention on a dependent variable over time [72, 73]. A quasi-experimental design, this approach is well-suited to real-world settings where randomized controlled trials cannot be conducted [74]. We begin with a time series, a sequence of observations indexed longitudinally by time. A real-world intervention (such as a policy change, natural disaster, or other major event) may alter a previously-established pattern in the data, creating a change point. Subsequences of data bounded by change points are called segments [72]. ITS requires at least 8 data points before and after the intervention in order to be well-defined [75]. Regression methods can be applied to each segment, provided the necessary assumptions are satisfied for that particular model. In this work, we use ITS with linear regression, also referred to as piecewise linear regression. Linear-regression-based ITS models have been used previously in opioid-related research, including analyses of utilization levels during a drug shortage [76], mortality rates after the introduction of overdose prevention and safe consumption sites [77], and emergency department visits and deaths surrounding harm-reduction interventions [78].

A simple ITS model takes on the form

$$\begin{array}{c} y_{t}=\beta_{0}+\beta_{1}t+\beta_{2}I_{t}+\beta_{3}P_{t}+\epsilon_{t}, \end{array}$$ (3)

where *y*_*t*_ is the outcome at time *t* relative to the starting point, $I_{t}$ is an indicator for whether the intervention has occurred, and *P*_*t*_ is the time elapsed since the intervention [79]. We adopt the convention that *P*_*t*_ = 0 before the intervention. The immediate effect *β*_2_ represents the instantaneous change in level due to the intervention. The sustained effect *β*_3_ is the change in slope with respect to time relative to the pre-intervention value.

We can also make use of the counterfactual

$$\begin{array}{c} {\tilde{y}}_{t}=\beta_{0}+\beta_{1}t, \end{array}$$ (4)

an extrapolation for what would have happened during the post-intervention time window in the absence of said intervention [80].

We can extend the ITS framework to formulate a model for multiple interventions,

$$\begin{array}{c} y_{t}=\underset{\text{pre-intervention}}{\underset{︸}{\beta_{0}+\beta_{1}t}}+\underset{\text{first}\;\text{intervention}}{\underset{︸}{\beta_{2}I_{t}^{\left(1\right)}+\beta_{3}P_{t}^{\left(1\right)}}}+\underset{\text{second}\;\text{intervention}}{\underset{︸}{\beta_{4}I_{t}^{\left(2\right)}+\beta_{5}P_{t}^{\left(2\right)}}}+\cdots +\underset{\text{last}\;\text{intervention}}{\underset{︸}{\beta_{2n}I_{t}^{\left(n\right)}+\beta_{2n+1}P_{t}^{\left(n\right)}}}+\epsilon_{t}, \end{array}$$ (5)

which is then applied to the opioid epidemic.

### Opioid epidemic timeline

We characterize the U.S. opioid epidemic through six defining events that constitute the interventions for our ITS analysis in the sections to follow. These six time points create five intervals between them, each of which may be referred to as a numbered segment (e.g. the “second segment” is the time window from the rise of heroin up until the rise of fentanyl).

#### Prescriptions

The underpinnings of the opioid crisis began in the 1980s, as studies advocated for the medical use of opioids in treating chronic pain whilst diminishing their addictive nature [81–83]. Increased prescriptions resulted in the production of new opioids, most notably Purdue Pharma’s sustained-release pain reliever OxyContin in 1996 [84]. This led to a rise in prescription misuse and overdose deaths. Since our data only begins in January 1999, we operate under the assumption that death rate trends from 1999 to 2010 (our next event) are a reasonable extension of 1996–1998. Conveniently, the plausibility of a lag time for OxyContin to become prevalent after its initial marketing makes 1999 a more robust starting point.

#### Heroin

Heroin, a recreational opioid typically injected intravenously, dominated the second wave of the opioid epidemic. Research shows increases in heroin use and dependence in the period of 2008 to 2011 compared to the early 2000s [85]. Graphical representations depict notable spikes in heroin death rates in 2010, consistent with CDC documentation [86, 87]. We set January 1, 2010 as the marker for the rise of heroin.

#### Fentanyl

The third wave was driven by synthetic opioids in 2013 [86]. Whilst fentanyl has medical uses as a potent pain reliever, illicit fentanyl and its myriad of derivatives have contributed to misuse and abuse, elevating opioid-related death rates [88]. Lack of regulation in a dynamic illicit drug market makes novel fentanyl compounds extremely dangerous, especially to those who have a history of opioid use and may, either knowingly or unknowingly, transition to such substances [89]. We set January 1, 2013 as the fentanyl event date.

#### Public health emergency

As a result of the highly addictive nature of opioids and the magnitude of the number of preventable deaths caused by opioid overdoses, the U.S. Department of Health and Human Services declared the opioid epidemic a PHE on October 26, 2017 [90]. National emergency status enables increased access to funding for treatment, and reinforces that necessary legal reforms must be made [91].

#### COVID-19 pandemic

The COVID-19 pandemic is an important epoch in the opioid epidemic timeline. Societal disruptions including social distancing restrictions, economic stress, and limited access to treatment disproportionately impact individuals with OUDs, increasing the risk of misuse leading to overdose [92]. We consider March 13, 2020, the date which COVID-19 was declared a national emergency in the U.S., as a proxy for the start of the impact of the pandemic on opioid-related deaths [93].

#### CDC funding

The COVID-19 pandemic catalyzed a myriad of knock-on effects including excess deaths due to various medical issues [94], mental health ramifications [95], and drug misuse/abuse [46], all of which were often exacerbated by health inequity [96]. On March 25, 2021, approximately a year into the pandemic, the CDC announced an initiative centered around community health in order to address both COVID-19- and non-COVID-19-related needs [97]. Encompassing improvements to physical and mental health care, programs may have been able to alleviate some of the death toll from opioid use. Whilst the funding itself was not issued to community health worker (CHW) organizations until September 2021, we can assume that such organizations would have been working on potential solutions as part of their applications to the funding. Hence, the funding announcement date of March 25, 2021 marks our final intervention [98].

### Notation

The aforementioned events are summarized in Table 1, where Variable Name is that used in R code and output, Variable Symbol denotes the mathematical representation throughout this paper, and Coefficient specifies the associated coefficient *β*_*i*_ in the ITS model. Though this model technically includes two public health emergency declarations, we use PHE to refer to that of the opioid epidemic.

**Table 1:** Reference table for ITS model notation.

Event / Intervention	Effect	Variable Name	Variable Symbol	Coefficient
Scope of analysis begins in January 1999	Immediate	(Intercept)	N/A	*β*_0_
Prescription opioids catalyze epidemic	Sustained	Month	*t*	*β*_1_
Heroin prevalence increases	Immediate	Indicator_Heroin	$I_{t}^{\left(\text{Heroin}\right)}$	*β*_2_
Heroin prevalence increases	Sustained	MonthsSince_Heroin	$P_{t}^{\left(\text{Heroin}\right)}$	*β*_3_
Fentanyl and synthetic opioids dominate	Immediate	Indicator_Fentanyl	$I_{t}^{\left(\text{Fentanyl}\right)}$	*β*_4_
Fentanyl and synthetic opioids dominate	Sustained	MonthsSince_Fentanyl	$P_{t}^{\left(\text{Fentanyl}\right)}$	*β*_5_
Opioid epidemic declared as PHE	Sustained	MonthsSince_PHE	$P_{t}^{\left(\text{PHE}\right)}$	*β*_6_
COVID-19 pandemic declared as emergency	Immediate	Indicator_COVID	$I_{t}^{\left(\text{COVID}\right)}$	*β*_7_
COVID-19 pandemic declared as emergency	Sustained	MonthsSince_COVID	$P_{t}^{\left(\text{COVID}\right)}$	*β*_8_
Funding announced for CHW services	Sustained	MonthsSince_CHW	$P_{t}^{\left(\text{CHW}\right)}$	*β*_9_

### Defining a model

Implementing Eq (5) with interventions of heroin, fentanyl, PHE declaration of the opioid epidemic, the COVID-19 pandemic, and CHW funding, we set up the following ITS regression model for monthly opioid-related death rate *y*_*t*_,

$$\begin{array}{c} \begin{array}{cc} y_{t}= & \beta_{0}+\beta_{1}t+\beta_{2}I_{t}^{\left(\text{Heroin}\right)}+\beta_{3}P_{t}^{\left(\text{Heroin}\right)}+\beta_{4}I_{t}^{\left(\text{Fentanyl}\right)}+\beta_{5}P_{t}^{\left(\text{Fentanyl}\right)}\\ & +\beta_{6}P_{t}^{\left(\text{PHE}\right)}+\beta_{7}I_{t}^{\left(\text{COVID}\right)}+\beta_{8}P_{t}^{\left(\text{COVID}\right)}+\beta_{9}P_{t}^{\left(\text{CHW}\right)}+\epsilon_{t}, \end{array} \end{array}$$ (6)

where the death rate is in units of deaths per 100,000 persons, and *t* measures time in months over the scope of our analysis. Note that each *P*_*t*_ term measures time in months from the specified event, thus we identify the monthly index associated with each event (Table 2), which is simply the number of months since January 1999 (month 1). Even though the PHE and CHW interventions occur late in the month, their lack of immediate effect in the model ensures the analysis is still robust. Additionally, said interventions may still have an impact on the last several days of the month, thus affecting the death rate no matter how slightly.

**Table 2:** Intervention dates with monthly indices.

Intervention	Date	Monthly Index
Heroin	1^st^ January, 2010	133
Fentanyl	1^st^ January, 2013	169
PHE	26^th^ October, 2017	226
COVID	13^th^ March, 2020	255
CHW	25^th^ March, 2021	267

We then compute indicators and elapsed times relative to each intervention, which generates explanatory variables:

$$\begin{array}{c} \begin{array}{cc} I_{t}^{\left(\text{Heroin}\right)}= & I\{t\geq 133\},\\ I_{t}^{\left(\text{Fentanyl}\right)}= & I\{t\geq 169\},\\ I_{t}^{\left(\text{PHE}\right)}= & I\{t\geq 226\},\\ I_{t}^{\left(\text{COVID}\right)}= & I\{t\geq 255\},\\ I_{t}^{\left(\text{CHW}\right)}= & I\{t\geq 267\} \end{array}\;\;\begin{array}{cc} P_{t}^{\left(\text{Heroin}\right)}= & \text{max}(0,P_{t}-132),\\ P_{t}^{\left(\text{Fentanyl}\right)}= & \text{max}(0,P_{t}-168),\\ P_{t}^{\left(\text{PHE}\right)}= & \text{max}(0,P_{t}-225),\\ P_{t}^{\left(\text{COVID}\right)}= & \text{max}(0,P_{t}-254),\\ P_{t}^{\left(\text{CHW}\right)}= & \text{max}(0,P_{t}-266), \end{array} \end{array}$$ (7)

where the indicator variables are calculated from the monthly indices in Table 2.

#### Interaction term

Whilst it is important to uncover trends in opioid-related death rates through the lens of critical time points, it is well-established that there are gender-based variations in opioid usage [99–101]. This can be modeled using an interaction term to augment the foundational ITS in Eq (6). The expressions for indicators and elapsed times remain as in Eq (7). Gender is a categorical variable which we code as Female = 0 and Male = 1, resulting in the following equation:

$$\begin{array}{c} \begin{array}{cc} y_{t}= & ({\hat{\beta }}_{0}+{\hat{\beta }}_{1}t+{\hat{\beta }}_{2}I_{t}^{\left(\text{Heroin}\right)}+{\hat{\beta }}_{3}P_{t}^{\left(\text{Heroin}\right)}+{\hat{\beta }}_{4}I_{t}^{\left(\text{Fentanyl}\right)}+{\hat{\beta }}_{5}P_{t}^{\left(\text{Fentanyl}\right)}\\ & +{\hat{\beta }}_{6}P_{t}^{\left(\text{PHE}\right)}+{\hat{\beta }}_{7}I_{t}^{\left(\text{COVID}\right)}+{\hat{\beta }}_{8}P_{t}^{\left(\text{COVID}\right)}+{\hat{\beta }}_{9}P_{t}^{\left(\text{CHW}\right)})\\ & +I\left\{\text{Gender}=\text{Male}\right\}({\overset{˘}{\beta }}_{0}+{\overset{˘}{\beta }}_{1}t+{\overset{˘}{\beta }}_{2}I_{t}^{\left(\text{Heroin}\right)}+{\overset{˘}{\beta }}_{3}P_{t}^{\left(\text{Heroin}\right)}+{\overset{˘}{\beta }}_{4}I_{t}^{\left(\text{Fentanyl}\right)}+{\overset{˘}{\beta }}_{5}P_{t}^{\left(\text{Fentanyl}\right)}\\ & \;\;\;\;+{\overset{˘}{\beta }}_{6}P_{t}^{\left(\text{PHE}\right)}+{\overset{˘}{\beta }}_{7}I_{t}^{\left(\text{COVID}\right)}+{\overset{˘}{\beta }}_{8}P_{t}^{\left(\text{COVID}\right)}+{\overset{˘}{\beta }}_{9}P_{t}^{\left(\text{CHW}\right)})\\ & +\epsilon_{t} \end{array} \end{array}$$ (8)

Eq (8) is based on the notion that female death rates act as the “baseline” in the model, whilst the interaction components govern the male death rates as deviations from the baseline. The $\left(\hat{\beta }+\overset{˘}{\beta }\right)$ terms illustrate this structure, where the $\hat{\beta }$ values represent the female model coefficients and the $\overset{˘}{\beta }$ values are modifications to those coefficients when considering the male population. Significant $\overset{˘}{\beta }$ coefficients indicate that the corresponding variable has differing effects between gender groups.

### Excess death rate and cumulative excess deaths

Excess death rates are computed as the difference between observed death rate and expected death rate. We forecast the expected opioid-related death rate ${\tilde{y}}_{t}$ through the counterfactual representing the hypothetical situation of the absence of the COVID-19 pandemic (and hence also the absence of the CHW intervention). Mathematically, this means $I_{t}^{\text{COVID}}$, $P_{t}^{\left(\text{COVID}\right)}$, and $P_{t}^{\left(\text{CHW}\right)}$ from Eq (6) are set to 0, resulting in a predictive formula of

$$\begin{array}{c} \begin{array}{cc} {\tilde{y}}_{t}= & \beta_{0}+\beta_{1}t+\beta_{2}I_{t}^{\left(\text{Heroin}\right)}+\beta_{3}P_{t}^{\left(\text{Heroin}\right)}+\beta_{4}I_{t}^{\left(\text{Fentanyl}\right)}+\beta_{5}P_{t}^{\left(\text{Fentanyl}\right)}+\beta_{6}P_{t}^{\left(\text{PHE}\right)}. \end{array} \end{array}$$ (9)

Note that Eq (9) can similarly be gender-stratified as above. We define the excess opioid-related death rate as

$$\begin{array}{c} r_{t}=y_{t}-{\tilde{y}}_{t}, \end{array}$$ (10)

where *y*_*t*_ represents the observed opioid-related death rate. From here, *r*_*t*_ is converted into excess opioid-related absolute deaths *s*_*t*_ using Eq (1) so that

$$\begin{array}{c} s_{t}=\frac{r_{t}p_{t}}{100000}. \end{array}$$ (11)

We then define *c*_*t*_ to represent cumulative excess absolute opioid-related deaths due to the pandemic at time *t* which yields

$$\begin{array}{c} c_{t}=\sum_{i=255}^{t}s_{i},\;t\geq 255, \end{array}$$ (12)

a quantity which only exists for months with the presence of the pandemic.

#### Bootstrapping

We obtain 95% bootstrapped confidence intervals (bsCI) for cumulative excess opioid-related deaths using simulations. This involves iterating through the following steps *B* times for large *B*, separately for each CR:

1. resample monthly opioid death counts according to $d_{t}^{*}∼\text{Poisson}\left(d_{t}\right)$ for all timepoints;
2. fit the appropriate ITS linear regression model (Eqs (6) or (8)) to the resampled data;
3. find the counterfactual predictions using the resampled data to get ${\tilde{y}}_{t}^{*}$;
4. calculate excess opioid-related absolute deaths $s_{t}^{*}$;
5. calculate cumulative excess opioid-related absolute deaths $c_{t}^{*}$ for each relevant timepoint.

For each timepoint *t* in a given CR, there are *B* simulations $c_{t,1}^{*},c_{t,2}^{*},\ldots ,c_{t,B}^{*}$ from which the 2.5 and 97.5 quantiles can be extracted in order to yield 95% confidence interval bounds. We utilize these results to examine national cumulative excess opioid-related deaths by summing $c_{t}^{*}$’s across CRs for each relevant *t* and then taking quantiles.

### Statistical analysis

Statistical analysis is performed using R version 4.3.0 [102], with packages dplyr, lubridate, ggplot2, and ggpubr [103–106]. Separately for each of the four CRs in the U.S., we run ITS regression for Eqs (6) and (8) using built-in linear modeling functionality, report model output and assess coefficients at the *α* = 0.05 significance level, and plot the regression line with both a 95% confidence interval and a 95% prediction interval. Then, with a modified dataset for March 2021 onwards where $I_{t}^{\left(\text{COVID}\right)}$, $P_{t}^{\left(\text{COVID}\right)}$, and $P_{t}^{\left(\text{CHW}\right)}$ are set to 0, we use the model to predict the counterfactual. The counterfactual is then plotted with a line for the fit and a shaded region for the 95% prediction interval. We run diagnostics for residuals versus fitted values, quantile-quantile (Q-Q) plot, Cook’s distance, and leverage in order to assess model assumptions and further interpret the results. Following that, we use the monthly opioid-related death rates from March 2020 onwards and the counterfactual in order to compute both the excess opioid-related death rate and the cumulative excess opioid-related deaths associated with the pandemic.

## Results

Statistical output from the ITS models allows us to discern how each key event impacted regional opioid-related death rates, as well as to infer any between-region similarities and differences. Excess deaths analysis then contributes towards quantifying the impact of COVID-19 on the opioid epidemic.

### Foundational model

**Fig 1:**
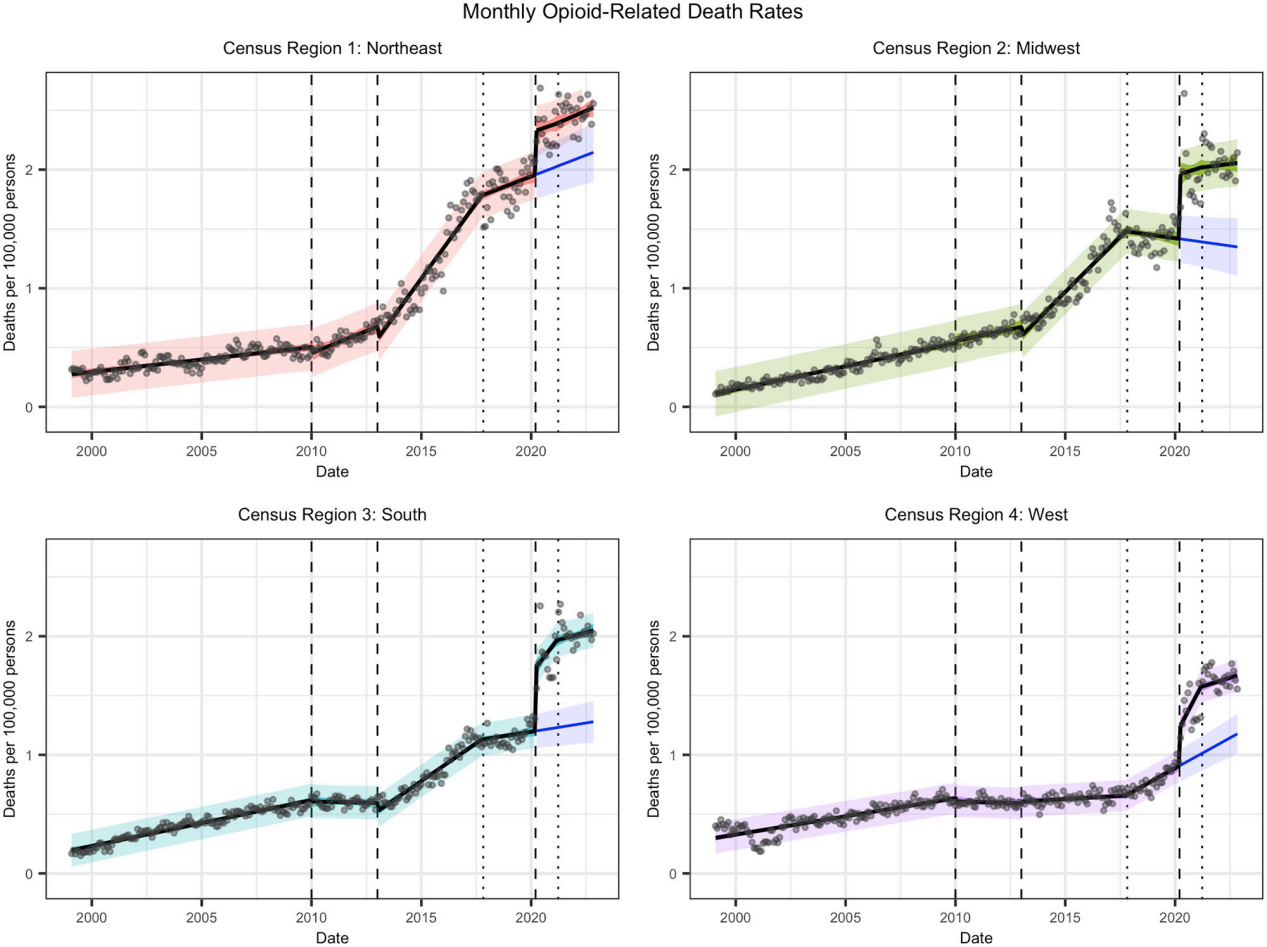
Monthly opioid-related death rates by CR for January 1999 to October 2022. Death rates are reported in units of deaths per 100,000 persons. Plot shows observed opioid-related death rates with superimposed ITS regression line, 95% confidence interval, and 95% prediction interval. The counterfactual representing the absence of the COVID-19 pandemic is also included in blue, with a trend line and 95% prediction interval. Vertical lines represent model interventions as described in our outline of the opioid epidemic.

We first discuss the results and diagnostics of the foundational model specified in Eq (6), focusing on the interpretation of coefficients from the statistical output. Fig 1 shows monthly opioid-related death rates along with the ITS regression line, 95% confidence interval, 95% prediction interval, and counterfactual in the absence of the COVID-19 pandemic, for each CR. Table 3 contains the estimated coefficients, standard errors, and *p*-values as per the model output. The residuals are symmetrically distributed, albeit according to a heavier-tailed distribution than a normal distribution, with mild heteroscedasticity in some CRs. There are a few influential points with high leverage in the pandemic segment, which is to be expected as this event shifted trends in opioid-related death rates.

**Table 3:** Statistical output for the foundational ITS regression model of monthly opioid-related death rates, January 1999 to October 2022.

	Northeast			Midwest			South			West		
Variable*	$\hat{\beta }$	SE	*p*-value	$\hat{\beta }$	SE	*p*-value	$\hat{\beta }$	SE	*p*-value	$\hat{\beta }$	SE	*p*-value
(Intercept)	0.2729	0.0172	< 0.001	0.1069	0.0167	< 0.001	0.1920	0.0121	< 0.001	0.2972	0.0115	< 0.001
*t*	0.0018	0.0002	< 0.001	0.0033	0.0002	< 0.001	0.0032	0.0002	< 0.001	0.0026	0.0001	< 0.001
$I_{t}^{\left(\text{Heroin}\right)}$	-0.0509	0.0376	0.177	0.0173	0.0365	0.6349	-0.01112	0.0265	0.673	-0.0263	0.0250	0.294
$P_{t}^{\left(\text{Heroin}\right)}$	0.0045	0.0016	0.006	0.0000	0.0015	0.9935	-0.0036	0.0011	0.002	-0.0031	0.0011	0.004
$I_{t}^{\left(\text{Fentanyl}\right)}$	-0.1043	0.0409	0.011	-0.0771	0.0397	0.0531	-0.0713	0.0288	0.014	0.0155	0.0272	0.570
$P_{t}^{\left(\text{Fentanyl}\right)}$	0.0150	0.0017	< 0.001	0.0122	0.0017	< 0.001	0.0110	0.0012	< 0.001	0.0014	0.0011	0.227
$P_{t}^{\left(\text{PHE}\right)}$	-0.0153	0.0020	< 0.001	-0.0177	0.0019	< 0.001	-0.0082	0.0014	< 0.001	0.0076	0.0013	< 0.001
$I_{t}^{\left(\text{COVID}\right)}$	0.3701	0.0648	< 0.001	0.5409	0.0628	< 0.001	0.5231	0.0456	< 0.001	0.3132	0.0431	< 0.001
$P_{t}^{\left(\text{COVID}\right)}$	-0.0006	0.0065	0.922	0.0070	0.0063	0.2655	0.0178	0.0046	< 0.001	0.0210	0.0043	< 0.001
$P_{t}^{\left(\text{CHW}\right)}$	0.0014	0.0085	0.874	-0.0029	0.0083	0.7254	-0.0160	0.0060	0.008	-0.0246	0.0057	< 0.001

* Symbols are as defined in Table 1.

**Northeast**: Residual standard error: 0.09843 on 276 degrees of freedom; Multiple R-squared: 0.9827; Adjusted R-squared: 0.9821; F-statistic: 1741 on 9 and 276 DF, p-value: < 0.001.

**Midwest**: Residual standard error: 0.09546 on 276 degrees of freedom; Multiple R-squared: 0.9755; Adjusted R-squared: 0.9747; F-statistic: 1222 on 9 and 276 DF, p-value: < 0.001

**South**: Residual standard error: 0.06934 on 276 degrees of freedom; Multiple R-squared: 0.9819; Adjusted R-squared: 0.9813; F-statistic: 1661 on 9 and 276 DF, p-value: < 0.001

**West**: Residual standard error: 0.06549 on 276 degrees of freedom; Multiple R-squared: 0.9647; Adjusted R-squared: 0.9635; F-statistic: 837.9 on 9 and 276 DF, p-value: < 0.001

#### Northeast

The defining moment of the opioid epidemic thus far is, undoubtedly, the introduction of fentanyl in the Northeast. Whilst the slope and intercept are both positive in the first segment, and heroin has a significant sustained effect, fentanyl drives an unprecedented monthly increase in opioid-related death rate, resulting in the greatest third segment slope across all CRs. The PHE appears to have the intended consequences with respect to abatement, with a significant negative sustained effect which counteracts the increases due to fentanyl from that point onwards. The COVID-19 pandemic clearly impacts the Northeastern opioid epidemic with a staggering immediate effect over pre-pandemic levels. Unfortunately, the CHW intervention has no significant effect on opioid-related death rates in this region, indicating that further effort is needed to address the crisis in the Northeast.

#### Midwest

The Midwest starts out with a lower initial opioid-related death rate and a higher growth rate compared to the Northeast, which may indicate a delay in the start of prediction opioids but a more rapid uptake subsequently. This is the only CR for which there are no statistically significant effects due to heroin. When fentanyl strikes at the sustained level, the magnitude is not quite that of the Northeast but still severe compared to the South and West. This may relate to the geographical proximity between the Midwest and Northeast and suggests possible illicit drug distribution patterns. An interesting feature of the fitted model is that the PHE yields a significant negative coefficient, indicating that control measures may have alleviated some of the burden from opioid-related deaths. However, we do note that the data points are somewhat curvilinear in this particular segment, decreasing at first before increasing in the leadup to the pandemic, which makes it tricky to discern a pattern. As a result, the statistically significant positive coefficient associated with the immediate impact of COVID-19 is likely an overestimate. Even so, a visual inspection of the data suggests that COVID-19 would have an extremely prominent immediate effect regardless of the trend in the PHE segment. As with the Northeast, the CHW intervention has no significant effect.

#### South

In the South, following the first segment, there is a significant sustained effect associated with heroin which is actually negative. This contrasts with the opioid-related death rate patterns observed in the first two regions, and could potentially signify earlier regional control measures in Southern states. In correspondence with the first two regions, fentanyl has a significant positive effect before the PHE reduces some of that impact. After the pandemic hits, we observe the immediate effect as expected, but also a significant sustained effect which was not observed for the Northeast or Midwest. This highlights a particular sensitivity to the nature of concurrent crises which may have catalyzed additional opioid-related deaths. With that said, the CHW intervention has a significant negative effect, which nearly counteracts the COVID-19 segment trend and starts to pull the slope nearer to being level.

#### West

We then turn to the West, which has an interesting epidemic trajectory compared to the other CRs. The significant negative sustained effect associated with heroin more than counteracts the rate of increase in the first segment to yield a negative slope until fentanyl enters the picture. However, unlike all other CRs, the West has no significant coefficients for fentanyl but a positive effect associated with the PHE segment. This indicates that the propagation of fentanyl in the West lagged behind the rest of the U.S., which lines up with existing research [107]. The regression line in the pre-pandemic segment has the highest slope across all CRs and is further exacerbated by the start of the pandemic. Whilst the slope returns to pre-pandemic levels after the CHW intervention, the immediate impact of COVID-19 resulted in a lasting impact on opioid-related death rates.

#### Excess deaths

**Fig 2:**
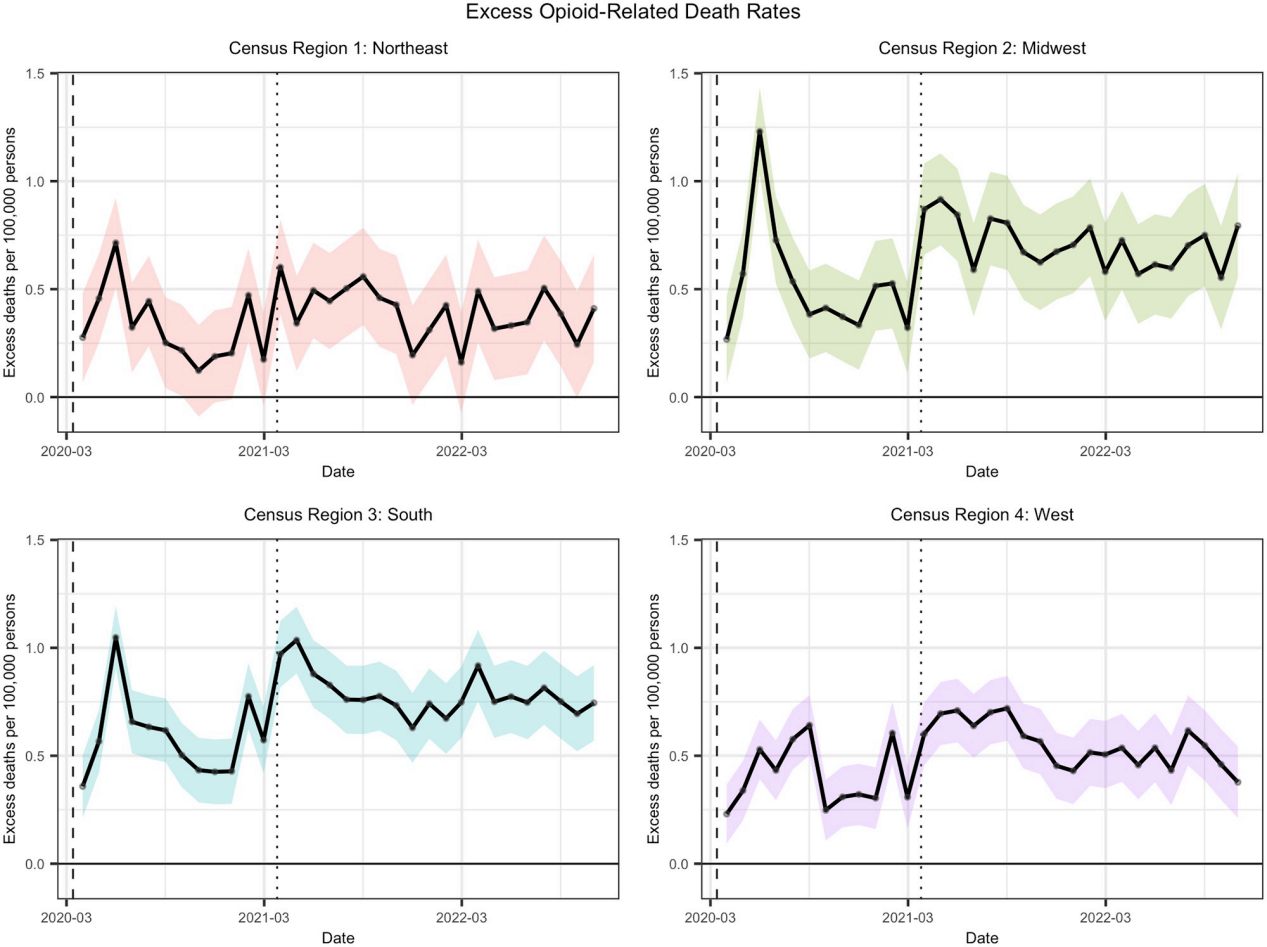
Excess opioid-related death rates by CR, March 2020 to October 2022. Death rates are reported in units of deaths per 100,000 persons. Black lines represent the excess opioid-related death rates calculated against the ITS counterfactual. Shaded regions represent a 95% prediction interval.

The results of the ITS model allow us to use the counterfactual regression line as a proxy for expected monthly death rates from March 2020 onwards in the absence of the pandemic. Fig 2 shows that, other than minimal overlaps of the 95% prediction interval with the *x*-axis seen in the Northeast, excess death rates are significantly positive. The Northeast, Midwest, and South all see peaks in May 2020, recording excess opioid-related death rates of 0.71 (95% PI 0.51–0.92), 1.23 (95% PI 1.03–1.43) and 1.05 (95% PI 0.90–1.19) (per 100,000 persons) respectively. In contrast, the West does not reach its maximum excess opioid-related death rate of 0.72 (95% PI 0.57–0.87) (per 100,000 persons) until August 2021, though a few of the preceding months report similar magnitudes in excess death rate. We also observe that excess opioid-related death rates in all regions appear to spike around March 2021, followed by a rough equilibrium in terms of distance from the counterfactual.

**Fig 3:**
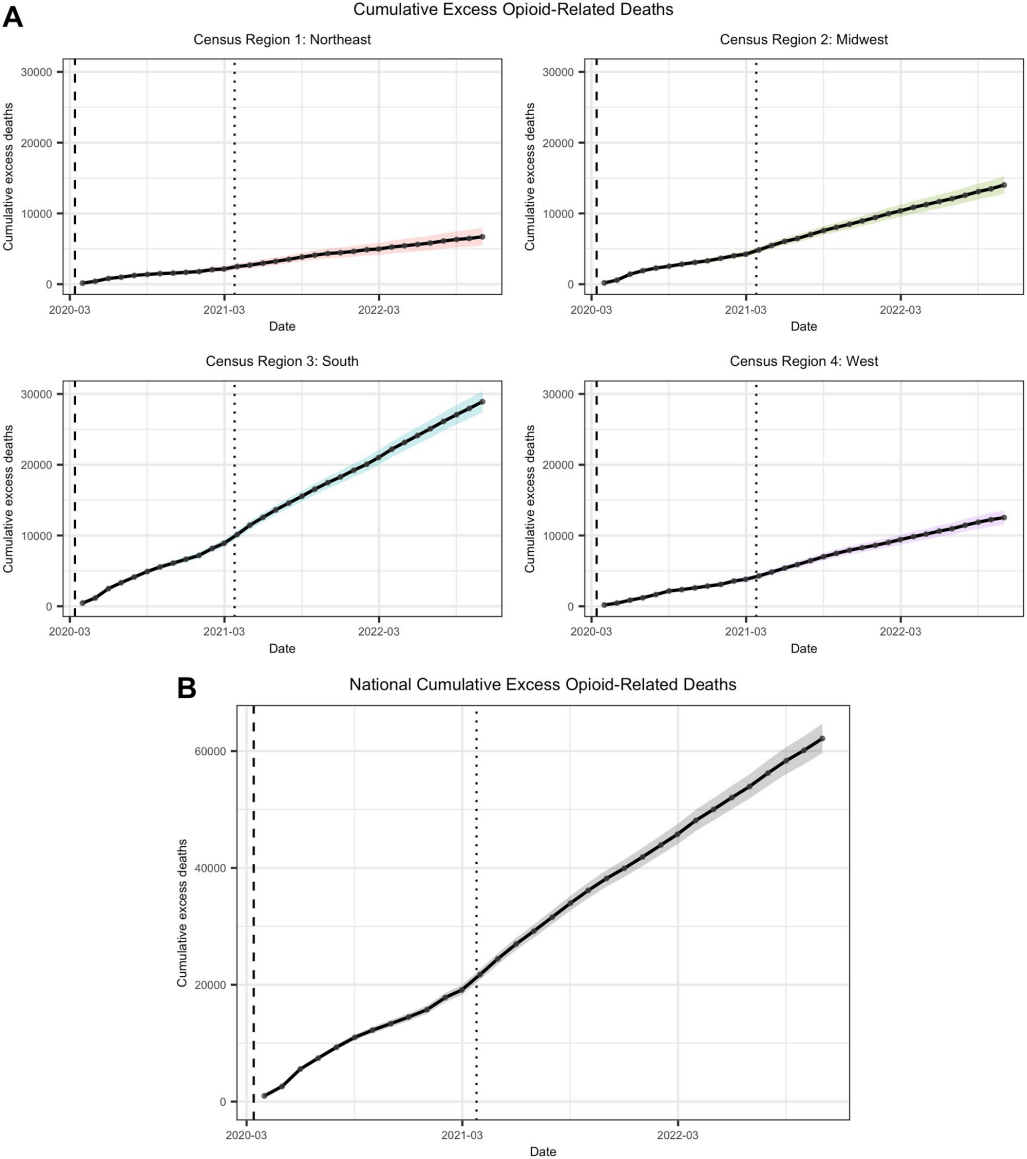
Cumulative excess opioid-related deaths (A) by CR and (B) nationally, March 2020 to October 2022. Black lines correspond to sums utilizing observed deaths compared to the ITS model predictions. Shaded regions represent 95% bootstrapped confidence intervals.

Switching focus to cumulative excess deaths (Fig 3), we can get a sense of the overall additional burden to the opioid-related death toll since the start of the pandemic. In just over two and a half years, spanning from March 2020 to October 2022, the estimated cumulative excess opioid-related deaths are 6,699 (95% bsCI 5,468–7,954), 14,015 (95% bsCI 12,817–15,231), 28,904 (95% bsCI 27,390–30,390), and 12,538 (95% bsCI 11,529–13,536) for the Northeast, Midwest, South, and West respectively. Aggregated to the national level, this quantity is 62,156 (95% bsCI 59,679–64,662).

We can contextualize these results with the literature on excess deaths induced by the pandemic. Numerous sources addressed all-cause cumulative excess deaths (aCED) associated with the pandemic, but we focus on the corresponding non-COVID-19 excess deaths (nCED) (e.g. deaths from drug overdoses, heart attacks, cancer) to capture the collateral damage. Since COVID-19 was not a cause of death prior to the pandemic,

$$\begin{array}{c} \text{nCED}=\text{aCED}-\text{COVID-19}\;\text{deaths}. \end{array}$$ (13)

Then, we evaluate the proportion of nCED attributable to opioids based on our model estimates of cumulative excess opioid-related deaths (oCED), where

$$\begin{array}{c} \text{Opioid}\;\text{Attr}\;\%=\frac{\text{oCED}}{\text{nCED}}. \end{array}$$ (14)

Our findings are shown in Table 4. Notably, during the two-year period following the start of the pandemic, approximately 15.8% (95% bsCI 15.2%–16.4%) of the nCED were attributable to opioids. There is also evidence that opioid-related deaths had the strongest contribution to the nCED early on over a short window, corresponding with the sudden severe impact of the pandemic on public well-being.

**Table 4:** Model-based estimates for the proportion of non-COVID-19 excess deaths attributable to opioids.

	Estimates* from the Literature				Model Estimates^†^	
Time Period	Source	aCED	Cov %	nCED	oCED	Opioid Attr %
March 1, 2020 to May 30, 2020	[108]	122,300	77.9%	27,065	5,582 (5,286–5,882)	20.6% (19.5%–21.7%)
January 26, 2020^‡^ to October 3, 2020	[109]	299,028	66.7%	98,679	12,230 (11,711–12,767)	12.4% (11.9%–12.9%)
March 1, 2020 to January 2, 2021	[110]	522,368	72.4%	144,174	15,724 (15,029–16,427)	10.9% (10.4%–11.4%)
March 1, 2020 to February 28, 2021	[111]	649,411	82.9%	111,049	19,102 (18,278–19,935)	17.2% (16.5%–18.0%)
March 1, 2020 to February 28, 2022	[112]	1,159,580	75.0%^§^	289,895	45,770 (44,059–47,507)	15.8% (15.2%–16.4%)

* Estimates correspond to exact values seen in the literature, or the mean value in cases where intervals were originally reported. All sources provided aCED values. Cov % represents the proportion of aCED attributable to COVID-19. nCED is calculated based on the count or percentage of COVID-19 deaths.

^†^ All model estimates are shown with 95% bsCIs.

^‡^ Note that our cumulative excess deaths calculations start from March 2020 onwards, so opioid-related calculations for this row will be underestimates.

^§^The proportion of excess deaths attributable to COVID-19 was not provided in [112], so we used the simple average of the preceding four values.

These statistics highlight the surges in opioid-related mortality over pre-pandemic levels, further warranting the urgent need for effective preventative measures. Even with regards to the overarching collateral damage from COVID-19 (i.e. excess deaths from causes other than COVID-19 itself), and keeping in mind that the literature-based estimates are pulled from various sources over different time periods, it is clear that the opioid epidemic contributed considerably to excess loss of life.

#### Summary

Whilst there is some commonality in the general trajectories of the opioid epidemic between CRs, such as the clear jump in monthly death rates associated with the COVID-19 pandemic and the widespread death rate increases due to fentanyl, we can also identify the varying impacts the interventions had. For example, heroin increased the slope of the death rate in the Northeast, but actually decreased it in the South and West, suggesting that there may have been early efforts to combat heroin-related mortality in the latter CRs. Additionally, funding for CHW services only had significant effects in the South and West; though these regions also generally reported lower monthly death rates throughout the opioid epidemic which could have facilitated the efficacy of such measures.

### Gender stratification

**Fig 4:**
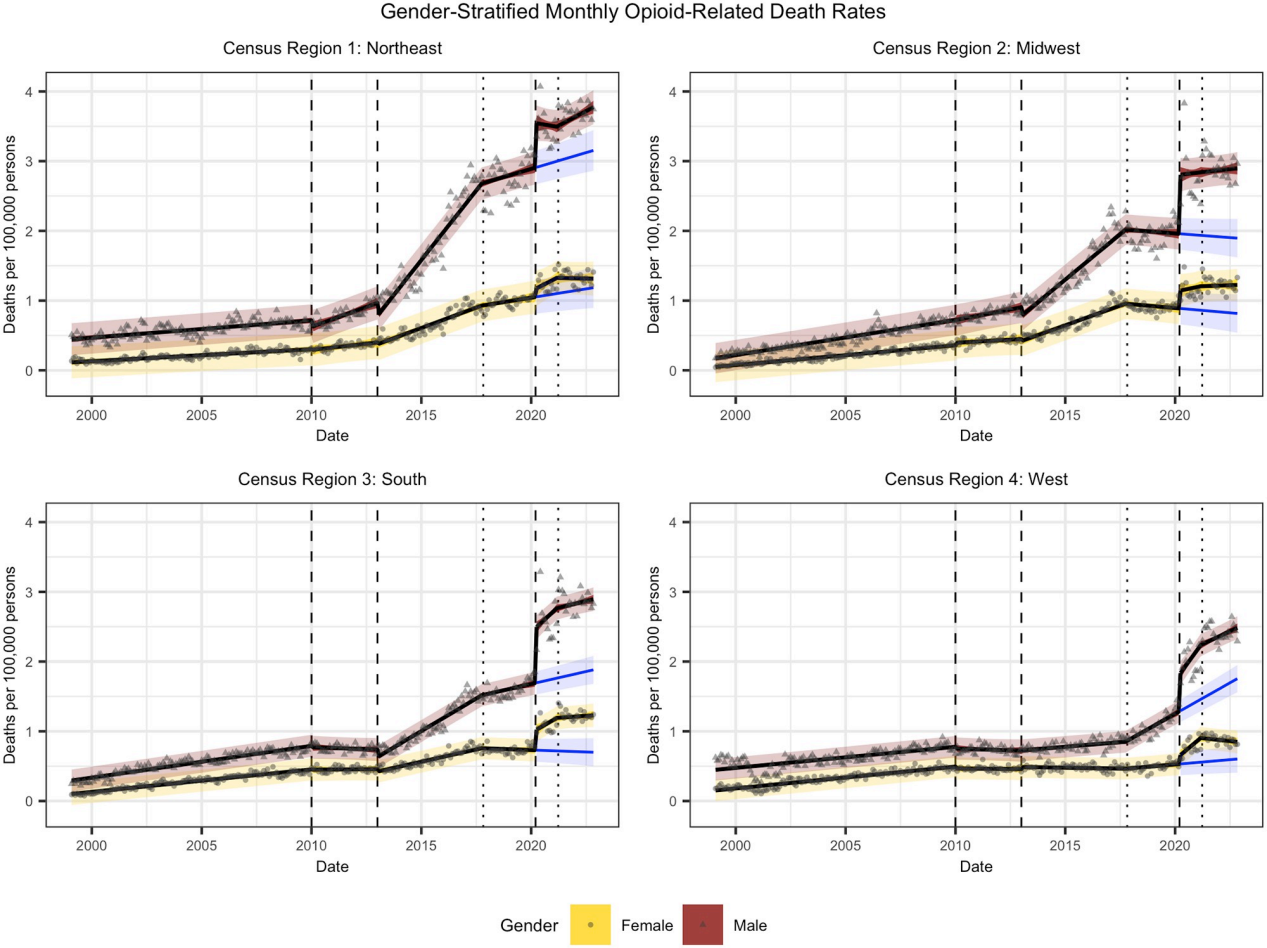
Gender-stratified monthly opioid-related death rates by CR for January 1999 to October 2022. Death rates are reported in units of deaths per 100,000 persons. Plot shows observed opioid-related death rates with superimposed ITS regression line, 95% confidence interval, and 95% prediction interval. The counterfactual representing the absence of the COVID-19 pandemic is also included in blue, with a trend line and 95% prediction interval. Vertical lines represent model interventions as described in our outline of the opioid epidemic.

We then refine our model with the interaction term for gender, as seen in Eq (8), and run identical analyses. The key idea here is to discern gender-based differences in opioid-related death rates and trends over the course of the epidemic. The accompanying plots are provided in Fig 4, whilst statistical output tables and detailed descriptions are contained in S2 Appendix. The residuals satisfy approximate normality, and heteroscedasticity is improved compared to the aggregated model. The high leverage and influential points are from male death rates, which demonstrate more variation particularly after the pandemic intervention.

The gender-stratified results confirm that the male demographic is much more vulnerable to opioid-related deaths. The sustained effect of fentanyl has significant positive interactions with gender in all CRs aside from the West. The immediate impact of COVID-19 is significant across all regions, as well as the gender interaction term. These coefficients corresponding to the pandemic are all positive, which means that whilst there were widespread impacts generally, the male demographic is much more vulnerable to opioid-related deaths. There is also more variation in post-COVID-19 male death rates (per 100,000 persons) between CRs, ranging from 2.5 in the West to 4 in the Northeast, whilst the female death rates (per 100,000 persons) hover close to 1.5 for all regions other than the West which largely remains below 1. The South has the largest pandemic-associated spike in opioid-related death rates for females compared to the other CRs, indicating potential underlying behavioral or societal differences in response to catastrophic events. Comparing the spikes in male and female opioid-related death rates associated with the onset of the pandemic, the greatest discrepancy occurs in the Midwest, followed closely by the Northeast, indicating an opportunity for carefully directed preventative care.

## Discussion

The primary goals of this work were to examine the impact of the COVID-19 pandemic on monthly opioid-related death rates in the four U.S. CRs, and identify possible factors driving the opioid epidemic on a broader timescale. We were also able to gain insight into regional and gender-based disparities, highlighting vulnerable demographies. Whilst the opioid epidemic still rages on in its fourth wave, these findings may provide additional points of consideration for developing effective measures to target the crisis. For example, developing safe injection sites in particularly hard-hit geographic regions [113, 114] or working to reduce stigma for vulnerable subpopulations [115–117] can be guided by our modeling results.

Through the baseline model of opioid-related death rates, we characterized the epidemic on a regional basis with regards to six key events. We note that the opioid epidemic has impacted the Northeast the most harshly, reaching monthly opioid-related death rates in excess of 2.5 deaths per 100,000 persons since the pandemic. The introduction of fentanyl and other illicit opioids in 2013 significantly changed the landscape, rendering previous policy approaches ineffective and leading to increases in fatal overdoses [118], which is clear from the 2013–2018 intervals in the Northeast, Midwest, and South. We also observed, on a national level, that the onset of the COVID-19 pandemic catalyzed unprecedented increases in opioid-related death rates, far above what would have been anticipated in the absence of the pandemic. The significant immediate upward shifts in opioid-related death rates for all regions speaks to how quickly and severely the impact of the pandemic stretched beyond the SARS-CoV-2 pathogen itself. It also raises the question as to whether other concurrent national or global catastrophes would also perturb the opioid epidemic in a similar manner, and what measures could possibly limit further mortality. For inevitable future pandemics and crises, it will be crucial to think about the collateral damage to other health and social services [119], particularly the opioid epidemic as the fourth wave continues to propagate [17, 18, 21, 120–122].

There is a clear disparity in opioid-related death rates between females and males. From the start of our analysis (January 1999), the male opioid-related death rates consistently surpass those seen amongst females, and further diverge as time goes on. Various research corroborates that males are more vulnerable to opioid overdose deaths than females [123, 124]. A retrospective analysis of opioid overdose deaths from 2016 to 2019 in Rhode Island showed that male deaths were more likely to have fentanyl exposure compared to female deaths, and that female deaths were more likely to be preceded by opioid prescriptions than male deaths [125], signifying that men may be skewed towards illicit opioid use. Data from British Colombia suggests that men using opioids alone and indoors contributes to a large proportion of overdose deaths [126], highlighting a particularly vulnerable subpopulation. Furthermore, a case study regarding a naloxone distribution program in Alaska revealed that female participants were more likely to engage in overdose prevention measures as opposed to males, who would tend to rely on naloxone [127]. This is evidence that any future intervention approaches need to be nuanced in order to be effective, and public health officials should take into consideration the gender-specific propensities for illicit drug use, addiction, and adherence to overdose prevention strategies.

It is important to keep in mind that these results must be interpreted retrospectively and in context of the scope of analysis. Regression methods do not lend themselves well to extrapolation outside of the scope of data, and so this is not a predictive endeavor. The advantage of our approach is that we were able to evaluate how different events and interventions impacted the opioid epidemic, which can directly aid in establishing robust measures that could be used in response to increasing opioid-related mortality. From our results and general literature, it is also clear that whilst COVID-19 greatly exacerbated opioid-related mortality, the increases in opioid-related mortality are also being driven systematically as opposed to solely being a knock-on effect of the pandemic. In other words, whilst COVID-19 greatly worsened its progression, the opioid epidemic was already a serious concern beforehand.

A paramount extension to this research lies in state-level analysis. Comparing states with similar pre-COVID-19 opioid-related death rate trajectories but different pandemic responses would indicate the effect these measures had on the opioid epidemic locally. Incorporating more demographic variables such as race, age, and income would also help identify vulnerable sub-populations and weaknesses in opioid management systems in the wake of concurrent large-scale health disasters. Additional region-specific interventions could also be incorporated for further realism in ITS models.

To gain further insight, the ICD-10 codes could be used to extract opioid deaths by substance or intent from the CDC WONDER database, the latter of which is largely unexplored and difficult to quantify as categorizations like intentional self-poisoning and assault require overwhelming evidence to declare. However, it is an important area given the known mental health ramifications of social distancing measures early in the pandemic [95]. A tangential study would examine relationships between opioid-related deaths and mental health during COVID-19 to uncover the psychological elements behind substance misuse and overdose. Regarding the former, existing research indicates that synthetic opioids like fentanyl are now the primary driver of the opioid epidemic, largely due to their potency and the frequency with which they are tweaked and incorporated into other substances [128]. The pandemic also may have contributed to a decrease in heroin, a change possibly driven by easier production and cheaper pricing of fentanyl making it a more viable option in COVID-19-induced economic struggles [129]. A deep dive into fentanyl-related mortality could help future-proof strategies for preventative measures as the nation grapples with the fourth wave.

## Conclusion

Through our research, we modeled the opioid epidemic with ITS methodology and showed that the COVID-19 pandemic did indeed alter the trajectory of the opioid epidemic with respect to monthly opioid-related death rates for all four CRs in the United States. Large immediate shifts in monthly opioid-related death rates associated with the pandemic suggest that the opioid epidemic is extremely sensitive to concurrent and severe external events. We also confirmed that opioid-related death rates are consistently greater amongst the male population, with the gap between genders further diverging in time.

Identifying vulnerable geographical regions and gender-specific variation behind fatalities is crucial for understanding and alleviating the opioid epidemic going forwards. The results from this work could help guide targeted, data-driven policies that address risk factors and create more robust treatment systems for OUD patients in order to reduce the scope of devastation, especially in instances of competing public health emergencies such as COVID-19. Without effective and timely preventative measures, the fourth wave will continue to sweep across the nation, bringing an unprecedented burden of opioid-related mortality in its wake.

## Supporting information

S1 Appendix U.S. Census Regions and Population. (PDF)

S2 Appendix Gender-stratified analysis. (PDF)

S3 Appendix Model diagnostics. (PDF)

S4 Appendix Sensitivity analyses. (PDF)
