# Supplementary material for: Evolution of an epidemic: Understanding the opioid epidemic in the United States and the impact of the COVID-19 pandemic on opioid-related mortality
Source: PLoS One. 2024 Jul 9;19(7):e0306395. doi: 10.1371/journal.pone.0306395 (PMC11233025; doi:10.1371/journal.pone.0306395)
Supplement: S1 Appendix — (PDF) [file pone.0306395.s001.pdf]

S1 Appendix

Demographics

Background information on U.S. CRs as per CDC WONDER [1] is shown in Table S1-1 (NB: a corresponding figure can be found from the U.S. Census Bureau [2]). Population sizes are displayed in Fig S1-1.

Table S1-1. U.S. States by Census Region.

Census Region	States
Northeast	CT, ME, MA, NH, NJ, NY, PA, RI, VT
Midwest	IL, IN, IA, KS, MI, MN, MO, NE, ND, OH, SD, WI
South	AL, AR, DE, DC, FL, GA, KY, LA, MD, MS, NC, OK, SC, TN, TX, VA, WV
West	AK, AZ, CA, CO, HI, ID, MT, NV, NM, OR, UT, WA, WY

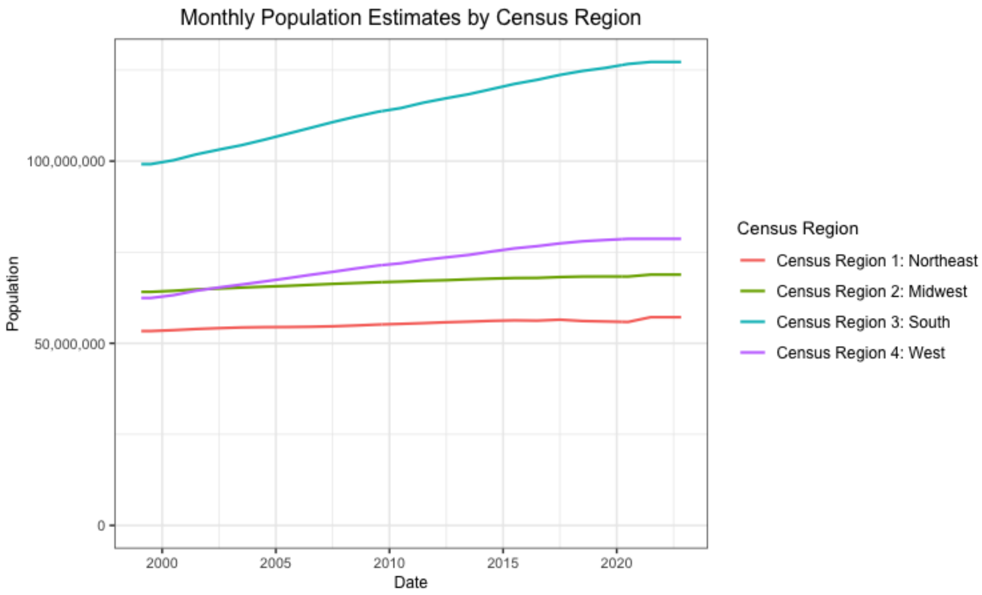

Fig S1-1. Monthly population size estimates for U.S. CRs, January 1999 to October 2022. Linearly interpolated from annual U.S. Census Bureau estimates.

Interpolation Method

As stated in the Data section, population estimates are provided on an annual basis, hence the need for interpolation in order to work with death rates at a monthly granularity. Linear interpolation, depicted in Fig S1-1, is a reasonable estimate under the assumption of continuous growth. An alternate option would be a step function, setting the population for each month of a given year to the annual estimate as seen in Fig S1-2. However, this regime leaves the death rates virtually unchanged (see Fig S1-3), with a maximum percent change of 1.2% for step interpolation relative to linear interpolation.

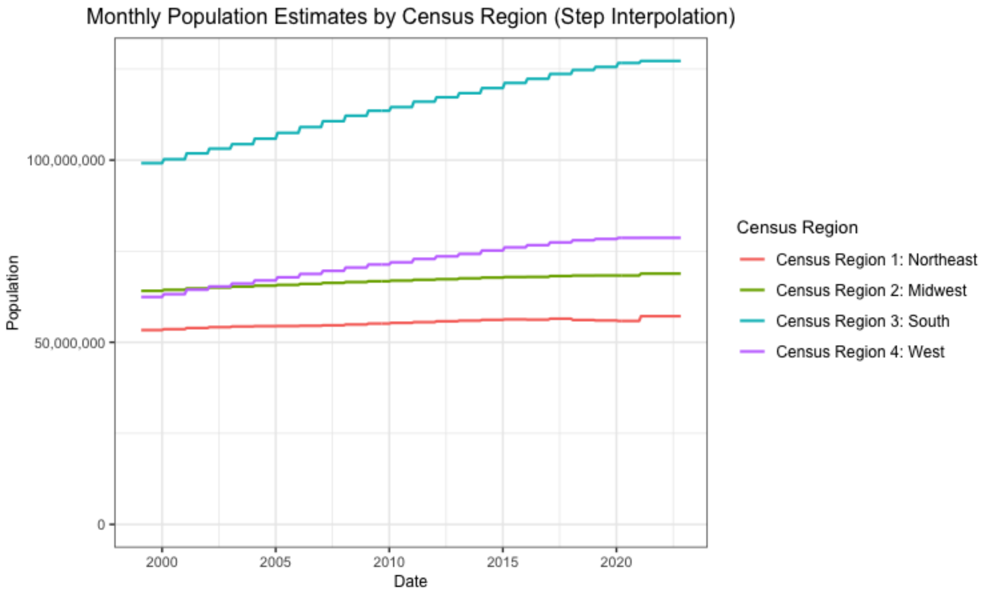

Fig S1-2. Alternate monthly population size estimates for U.S. CRs, January 1999 to October 2022. Step-function interpolated from annual U.S. Census Bureau estimates.

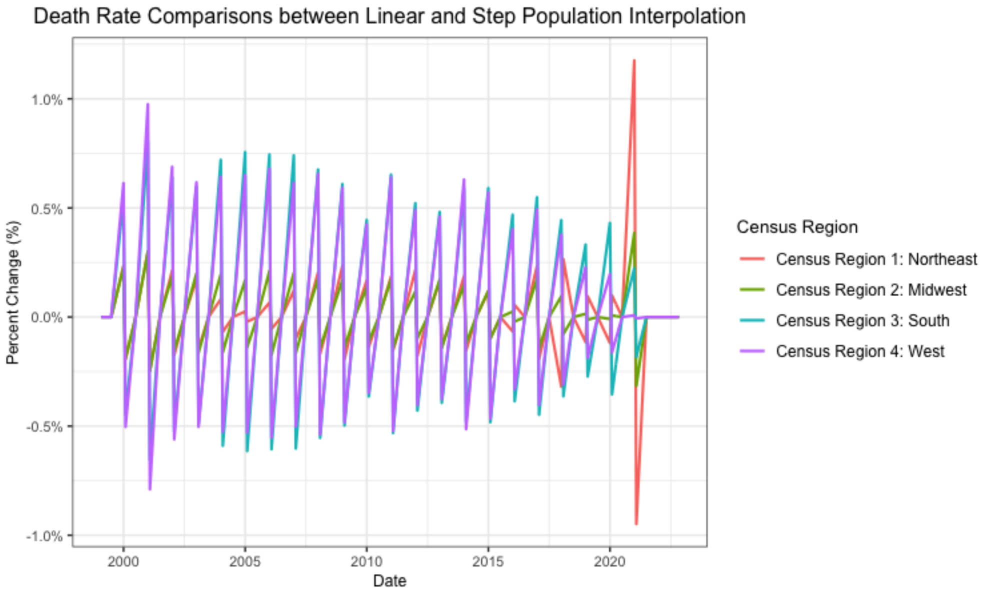

Fig S1-3. Monthly death rate is robust to the interpolation method used for estimating population. Percent change in death rate between linear and step interpolation methods for monthly population estimates, calculated as $\frac{y_{\text{step}} - y_{\text{linear}}}{y_{\text{linear}}}$. Oscillations with annual periodicity are present, and the 0% change occurs when populations are equivalent in July.

Population estimates come from the ground truth where COVID-19 has occurred, as do the death counts which we use to calculate the death rates that constitute the dependent variable in our model. When we make projections to the death rate in the but-for world (absence of COVID-19), it is a continuation of the pre-COVID-19 trend in which the underlying monthly opioid death counts and annual population estimates would have been growing in a pre-existing manner, directly contributing to the death rates. Hence, the counterfactual projections do not actually rely on any population estimates. Since excess death rate is calculated as observed death rate minus counterfactual death rate, and we have shown that the results are robust to interpolation method, the calculation of excess death rate is reasonable.

A deeper dive into cumulative excess opioid-related deaths estimates (denoted as "oCED") with 95% bsCIs can be found in Table S1-2, with a visual representation in Fig S1-4. The percent change (denoted as "%diff") for the mean oCED under step interpolation versus linear interpolation is calculated as

$$\%\text{diff} = \left(\frac{\text{oCED}_{\text{Step}} - \text{oCED}_{\text{Linear}}}{\text{oCED}_{\text{Linear}}} \right) \times 100.$$

With the magnitude of all %diff values being under 0.25%, we conclude that the population interpolation method is not influential on the results.

Table S1-2. Comparisons for estimates of cumulative excess opioid-related deaths by population interpolation method, March 2020 to October 2022.

Group		Linear Interpolation	Step Interpolation	
Region	Gender	oCED	oCED	%diff
Northeast	–*	6,699 (5,468–7,954)	6,713 (5,482–7,968)	0.2%
Midwest	–	14,015 (12,817–15,231)	14,025 (12,827–15,242)	0.1%
South	–	28,904 (27,390–30,390)	28,884 (27,369–30,370)	-0.1%
West	–	12,538 (11,529–13,536)	12,538 (11,529–13,537)	0.0%
National	–	62,156 (59,679–64,662)	62,160 (59,684–64,665)	0.0%
Northeast	Female	1,617 (987–2,254)	1,620 (990–2,257)	0.2%
Midwest	Female	3,869 (3,182–4,540)	3,871 (3,185–4,541)	0.1%
South	Female	9,463 (8,621–10,314)	9,455 (8,612–10,308)	-0.1%
West	Female	3,434 (2,883–3,984)	3,433 (2,882–3,984)	-0.0%
National	Female	18,382 (17,017–19,761)	18,379 (17,013–19,758)	-0.0%
Northeast	Male	5,006 (3,919–6,056)	5,018 (3,931–6,067)	0.2%
Midwest	Male	10,098 (9,095–11,080)	10,107 (9,105–11,089)	0.1%
South	Male	19,358 (18,114–20,609)	19,349 (18,104–20,600)	-0.0%
West	Male	9,057 (8,223–9,885)	9,059 (8,225–9,887)	0.0%
National	Male	43,520 (41,467–45,594)	43,533 (41,481–45,607)	0.0%

* Groups with a "–" in the Gender column signify results from the aggregate model as opposed to the gender-stratified model.

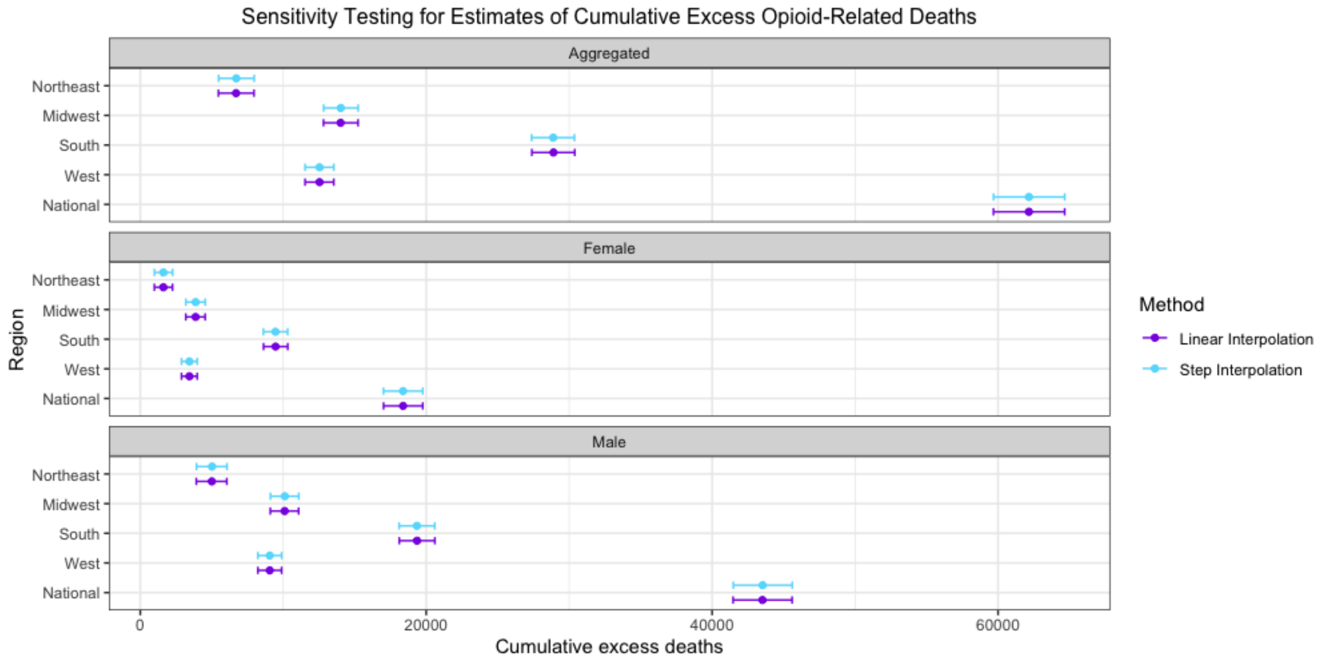

Fig S1-4. 95% bsCIs for estimates of cumulative excess opioid-related deaths by population interpolation method, March 2020 to October 2022.
Intervals are grouped by region and panelled by gender.

References

1. Centers for Disease Control and Prevention. Multiple Cause of Death by Single Race 2018-2022: Census Regions; 2024. Available from: `https://wonder.cdc.gov/wonder/help/mcd-expanded.html#Census%20Regions`.

2. United States Census Bureau. Census Regions and Divisions of the United States; 2024. Available from: `https://www2.census.gov/geo/pdfs/maps-data/maps/reference/us_regdiv.pdf`.
