# Supplementary material for: Evolution of an epidemic: Understanding the opioid epidemic in the United States and the impact of the COVID-19 pandemic on opioid-related mortality
Source: PLoS One. 2024 Jul 9;19(7):e0306395. doi: 10.1371/journal.pone.0306395 (PMC11233025; doi:10.1371/journal.pone.0306395)
Supplement: S2 Appendix — (PDF) [file pone.0306395.s002.pdf]

S2 Appendix

Gender-Stratified Analysis

Table S2-1 contains statistical output from the gender-stratified model for each of the four CRs. Recall that the model relates opioid-related death rates to time and the presence of various intervention events, and that death rates are reported in units of deaths per 100,000 persons.

Table S2-1. Statistical output for the gender-stratified ITS regression model of monthly opioid-related death rates, January 1999 to October 2022.

	**Northeast**			**Midwest**			**South**			**West**		
Variable	$\hat{\beta}$	SE	p-value	$\hat{\beta}$	SE	p-value	$\hat{\beta}$	SE	p-value	$\hat{\beta}$	SE	p-value
`(Intercept)`	0.1135	0.0202	< 0.001	0.0473	0.0192	0.014	0.0990	0.0139	< 0.001	0.1495	0.0136	< 0.001
t	0.0014	0.0003	< 0.001	0.0024	0.0003	< 0.001	0.0027	0.0002	< 0.001	0.0026	0.0002	< 0.001
$\mathbb{I}_t^{(\text{Heroin})}$	-0.0107	0.0441	0.809	0.0361	0.0418	0.388	-0.0032	0.0304	0.917	-0.0270	0.0296	0.362
$P_t^{(\text{Heroin})}$	0.0016	0.0019	0.407	-0.0009	0.0018	0.618	-0.0025	0.0013	0.055	-0.0028	0.0013	0.025
$\mathbb{I}_t^{(\text{Fentanyl})}$	-0.0269	0.0480	0.575	-0.0290	0.0455	0.525	-0.0343	0.0331	0.301	0.0291	0.0323	0.367
$P_t^{(\text{Fentanyl})}$	0.0067	0.0023	< 0.001	0.0079	0.0019	< 0.001	0.0057	0.0014	< 0.001	-0.0002	0.0014	0.885
$P_t^{(\text{PHE})}$	-0.0055	0.0023	0.019	-0.0117	0.0022	< 0.001	-0.0068	0.0016	< 0.001	0.0027	0.0016	0.090
$\mathbb{I}_t^{(\text{COVID})}$	0.1134	0.0760	0.136	0.2497	0.0721	< 0.001	0.2785	0.0524	< 0.001	0.1093	0.0511	0.033
$P_t^{(\text{COVID})}$	0.0093	0.0076	0.223	0.0079	0.0072	0.276	0.0162	0.0052	0.002	0.0198	0.0051	< 0.001
$P_t^{(\text{CHW})}$	-0.0140	0.0100	0.161	-0.0046	0.0095	0.626	-0.0134	0.0069	0.053	-0.0249	0.0067	< 0.001
`M`	0.3301	0.0286	< 0.001	0.1219	0.0271	< 0.001	0.1900	0.0197	< 0.001	0.2958	0.0192	< 0.001
`M:`t	0.0002	0.0004	0.096	0.0018	0.0004	< 0.001	0.0011	0.0003	< 0.001	-0.0001	0.0002	0.723
`M:`$\mathbb{I}_t^{(\text{Heroin})}$	-0.0825	0.0624	0.186	-0.0380	0.591	0.521	-0.0163	0.0430	0.705	0.0014	0.0419	0.973
`M:`$P_t^{(\text{Heroin})}$	0.0060	0.0026	0.025	0.0018	0.0025	0.470	-0.0022	0.0018	0.222	0.0005	0.0018	0.784
`M:`$\mathbb{I}_t^{(\text{Fentanyl})}$	-0.1587	0.0679	0.020	-0.0975	0.0644	0.131	-0.0754	0.0468	0.108	-0.0271	0.0456	0.552
`M:`$P_t^{(\text{Fentanyl})}$	0.0170	0.0029	< 0.001	0.0087	0.0027	0.001	0.0108	0.0020	< 0.001	0.0032	0.0019	0.097
`M:`$P_t^{(\text{PHE})}$	-0.0200	0.0033	< 0.001	-0.0121	0.0031	< 0.001	-0.0024	0.0023	0.225	0.0099	0.0022	< 0.001
`M:`$\mathbb{I}_t^{(\text{COVID})}$	0.5303	0.1075	< 0.001	0.5927	0.1019	< 0.001	0.5021	0.0741	< 0.001	0.4109	0.0722	< 0.001
`M:`$P_t^{(\text{COVID})}$	-0.0216	0.0108	0.045	-0.0027	0.0102	0.794	0.0024	0.0074	0.746	0.0016	0.0072	0.820
`M:`$P_t^{(\text{CHW})}$	0.0325	0.0141	0.022	0.0043	0.0134	0.750	-0.0048	0.0097	0.624	0.0010	0.0095	0.912

Northeast: Residual standard error: 0.1155 on 552 degrees of freedom; Multiple R-squared: 0.9853; Adjusted R-squared: 0.9848; F-statistic: 1942 on 19 and 552 DF, p-value: < 0.001.
Midwest: Residual standard error: 0.1095 on 552 degrees of freedom; Multiple R-squared: 0.9773; Adjusted R-squared: 0.9765; F-statistic: 1249 on 19 and 552 DF, p-value: < 0.001.
South: Residual standard error: 0.07964 on 552 degrees of freedom; Multiple R-squared: 0.9836; Adjusted R-squared: 0.9830; F-statistic: 1748 on 19 and 552 DF, p-value: < 0.001.
West: Residual standard error: 0.07758 on 552 degrees of freedom; Multiple R-squared: 0.9709; Adjusted R-squared: 0.9699; F-statistic: 970.6 on 19 and 552 DF, p-value: < 0.001.

Northeast

Compared to the aggregated model for the Northeast, the only significant coefficients at the baseline level are the intercept, month, sustained effect of fentanyl, and sustained effect of the PHE. However, all of the interaction terms aside from month and immediate effect of heroin are significant, which highlights strong evidence for gender discrepancies. These interactions are largely positive; in particular the contributions to the death rate from the male gender alone, the interaction with the sustained effect of fentanyl, and the interaction with the immediate effect of COVID-19 drive substantial growth in male opioid-related death rates. One notable coefficient is the sustained effect associated with the pandemic, where the negative interaction term suggests that the opioid-related death rates for the female demographic may have been more strongly impacted.

Midwest

In the Midwest, the set of variables with significant coefficients is identical amongst the baseline and interaction terms. These are specifically for the intercept, time, sustained effects of fentanyl and the PHE, and the immediate effect of COVID-19. This tells us that both genders responded to the same events on the same timescales, but at different magnitudes. As seen in the Northeast and in line with prior knowledge, the interaction terms tend to result in augmented death rates amongst males. The one exception to this is the negative interaction term associated with the sustained effect of the PHE, which suggests that males may have responded better to any initiatives implemented in the region.

South

Based on the interaction terms, opioid-related death rates amongst the female and male demographics in the South have similar responses to heroin, but then diverge with the introduction of fentanyl which catalyzes male mortality. Similarly to the Midwest, the gender interaction is significant for the immediate impact of COVID-19 but not the sustained effect, signifying that male death rates were much more sensitive to the onset of the pandemic.

West

The West records the most similarity between the opioid-related death rate trajectories between males and females, with the only significant gender interactions resulting from the intercept, the sustained effect of the PHE, and the immediate effect of COVID-19. The positive effect from the PHE observed in foundational model can now be attributed to increasing death rates within the male demographic, likely due to the delayed arrival of fentanyl in the region, suggesting that efforts in combating opioid-related deaths need to be focused on fentanyl use patterns in males.

Excess Deaths

As seen in Fig S2-1, after stratifying for gender, the mean predicted excess opioid-related death rates are greater than 0 for every census-region and gender combination (including for the female population in the Northeast, which records a minimum death rate of 0.004 (95% PI -0.27–0.27) (per 100,000 persons) in February 2022). Males are disproportionately impacted by the opioid crisis which is reflected in the figures. With that said, there are extremely similar patterns between male and female excess death trajectories, which indicates that both genders were impacted similarly by the pandemic in directionality but not in magnitude. The maximum female and male maximum excess death rates for the Midwest sync up in May 2020 at 0.88 (95% PI 0.65–1.11) and 1.88 (95% PI 1.65–2.11) (per 100,000 persons) respectively, but all other CRs have offset peaks. Interestingly, the male populations in the Northeast and South both experience peaks in May 2020 whilst the female populations in these CRs do not reach their peaks until March 2021. On the other hand, the Western plot does not display a distinct peak pattern. The gender-stratified cumulative absolute excess deaths plots in Fig S2-2 show that the large majority of deaths originate from the male population. In the Northeast, Midwest, South, and West, the final mean estimated female cumulative excess death tolls are 1,617 (95% bsCI 987–2,254), 3,869 (95% bsCI 3,182–4,540), 9,463 (95% bsCI 8,621–10,314), and 3,434 (95% bsCI 2,883–3,984) respectively. For males, these calculations result in cumulative opioid-related excess deaths of 5,006 (95% bsCI 3,919–6,056), 10,098 (95% bsCI 9,095–11,080), 19,358 (95% bsCI 18,114–20,609), and 9,057 (95% bsCI 8,223–9,885). Of the estimated 62,156 (95% bsCI 59,679–64,662) cumulative excess opioid-related deaths which occurred nationally from March 2021 to October 2022, 43,520 (95% bsCI 41,467–45,594) are attributable to males. There are no overlaps in bsCIs within each CR, further emphasizing the demographic differences underpinning opioid-related mortality.

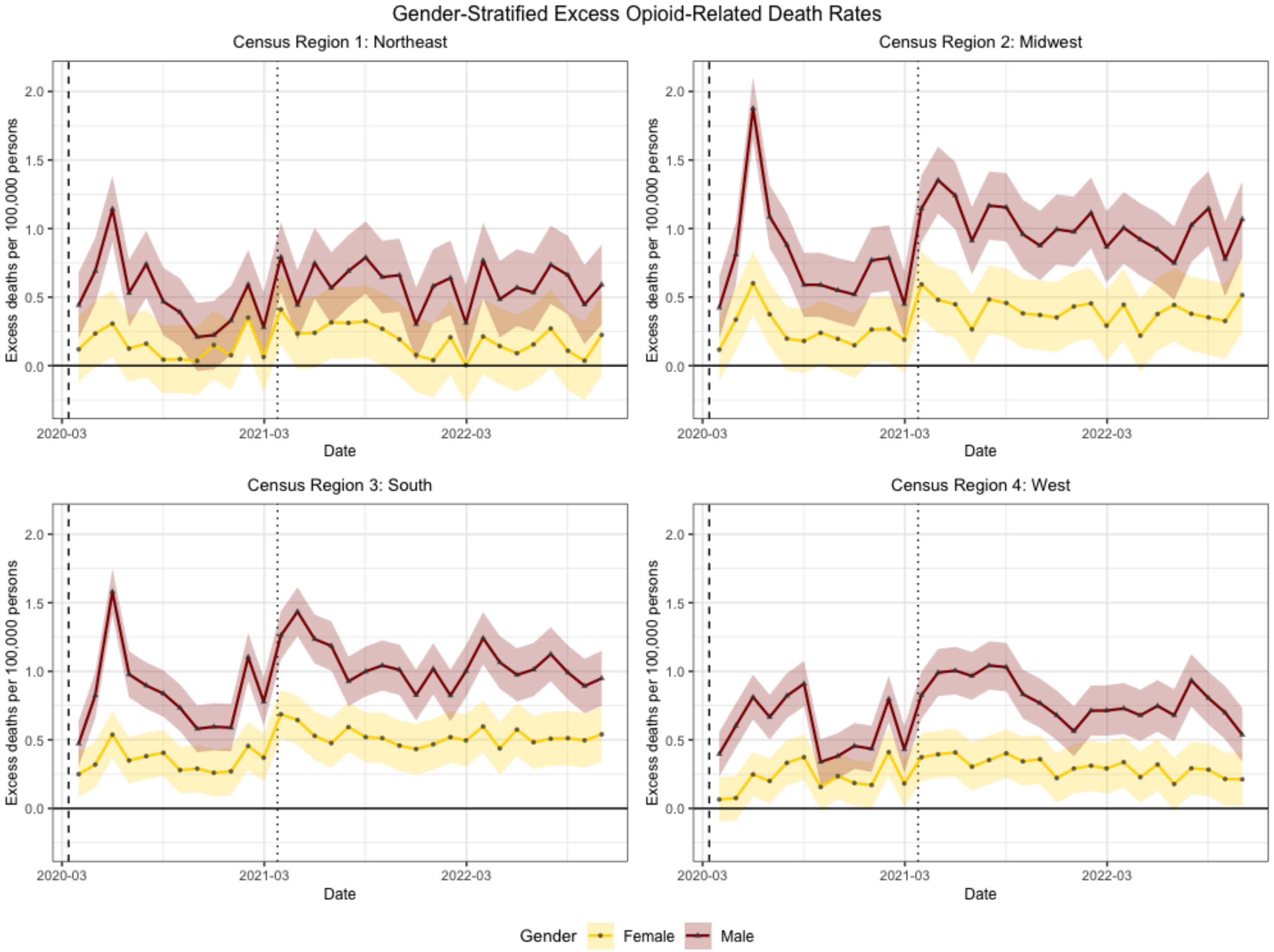

Fig S2-1. Excess opioid-related death rates by CR, March 2020 to October 2022. Solid lines represent the excess opioid-related death rates calculated against the ITS counterfactual. Shaded regions represent a 95% prediction interval.

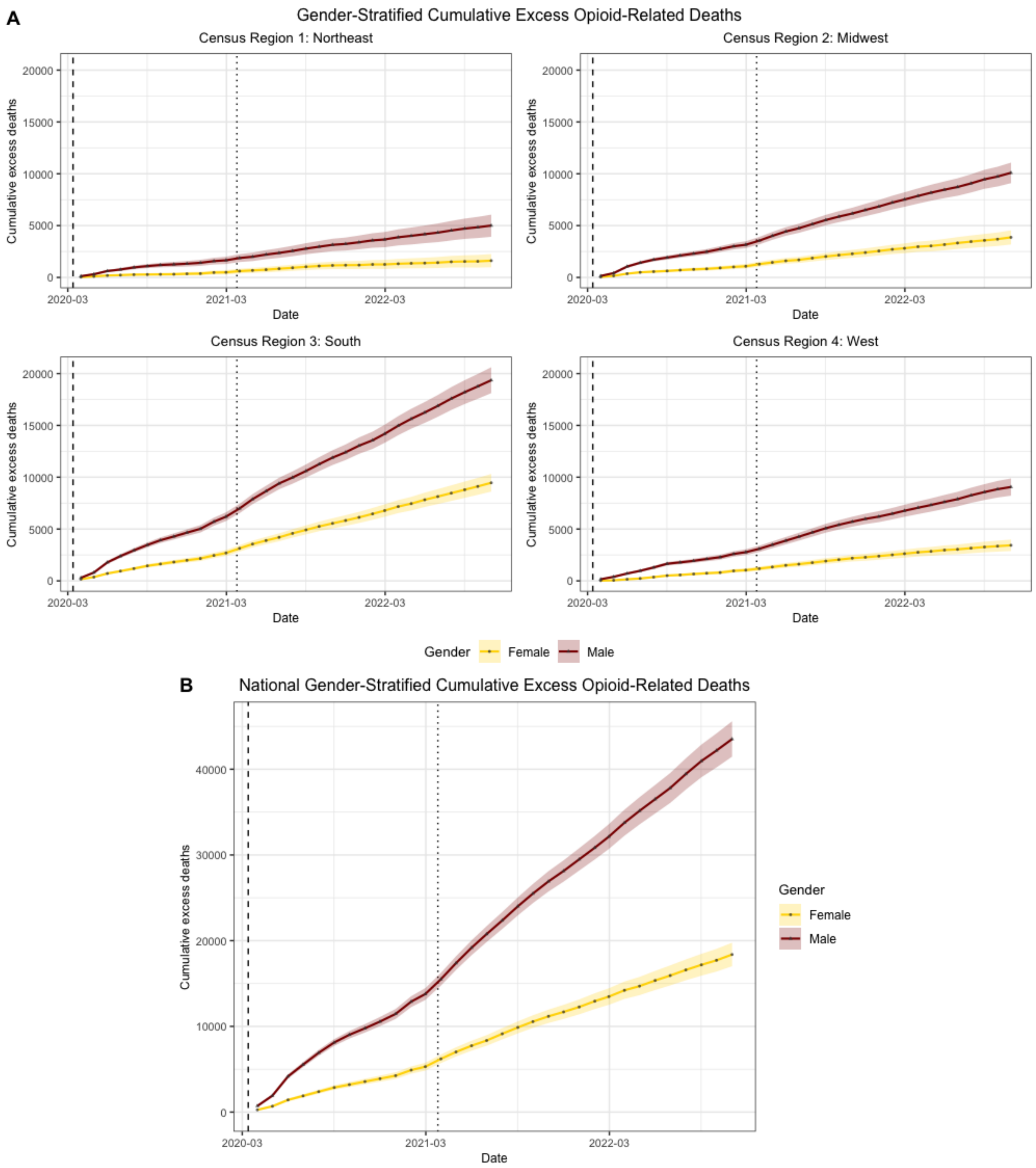

Fig S2-2. Cumulative excess opioid-related absolute deaths (A) by CR and (B) nationally, March 2020 to 2022.
Solid lines correspond to sums of excess deaths computed by comparing observed deaths to predicted deaths obtained from the gender-stratified ITS model predictions. Shaded regions represent 95% bootstrapped confidence intervals.
