# Supplementary material for: Evolution of an epidemic: Understanding the opioid epidemic in the United States and the impact of the COVID-19 pandemic on opioid-related mortality
Source: PLoS One. 2024 Jul 9;19(7):e0306395. doi: 10.1371/journal.pone.0306395 (PMC11233025; doi:10.1371/journal.pone.0306395)
Supplement: S3 Appendix — (PDF) [file pone.0306395.s003.pdf]

S3 Appendix

Model Diagnostics

Foundational Model

We now work through diagnostics for an ITS model with linear regression to check whether the necessary assumptions are satisfied for the models in each region. Fig S3-1 shows standard plots addressing linearity, homoscedasticity, normality, and influential observations. Fig S3-2 shows autocorrelation and partial autocorrelation which addresses independence of observations.

Residuals. A plot of residuals vs fitted values enables us to check linearity and homoscedasticity. We hope to observe an even split of residuals about zero. For the Northeast, the residual plot demonstrates a fairly random scatter about zero, though there is a dip in average residuals around fitted values of 1, followed by a slight increasing pattern corresponding with the third segment (fentanyl to PHE) data being slightly more curved rather than linear. The Midwest and South average residuals lines stay close to the horizontal in support of linearity, though both show an extreme residual for May 2020. All three of those regions show moderate heteroscedasticity, with lower variation amongst smaller fitted values and greater variation amongst larger fitted values, in line with increasing variability over the course of the opioid epidemic. The West satisfies homoscedasticity, with lower variation at the higher end of fitted values compared to the rest of the nation. There is a positive bias to the residuals at low fitted values, but overall linearity is satisfied as with the other regions. All plots have a gap in fitted values, matching the interrupted time series structure and significant immediate shifts in death rate.

Normal Q-Q. The data are consistent with normally distributed errors if the scatterplot depicts a pattern close to $y = x$. The Northeastern and Western normal Q-Q plots look appropriate for a symmetric distribution with heavier tails than a standard normal, given the behavior outside theoretical quantile interval $(-1.5, 1.5)$. The Midwest and South both have May 2020 as a data point with a standardized residual around 8, but the overarching shape of the remaining points behaves similarly to the aforementioned two. There are no severe violations of normality.

Cook's Distance. Cook's distance measures the influence of a data point on the regression model. Points with Cook's distances exceeding $C_k = \frac{8}{n-2p}$, where n and p are the numbers of observations and parameters involved in each regression, must be investigated further. In our case, $C_k = \frac{8}{286-2(10)} \approx 0.030$. The Midwest, South, and West all have a distinct rise in Cook's distances from index 250 onwards, corresponding with the COVID intervention. For the Northeast this rise occurs around index 200, encompassing the tail end of the fentanyl segment to present where the death rates demonstrate lots of variability. May 2020 is a high influence point over all locations, corresponding with the opioid death rate spikes. However, it would not be appropriate to remove these points as contrasting behaviors to previous death rate patterns are expected under a pivotal event such as COVID-19.

Leverage. Points with large Cook's distances themselves are not necessarily a concern, but when paired with high leverage they may have strong impacts on the model fit. The leverage plots show curves for extreme Cook's distances, so any points beyond those thresholds could be problematic. We note that March 2020 comes close to the 0.5-threshold and May 2020 exceeds the 1-threshold in the Midwest and South, but all other points fall within the bounds and are not cause for concern. Additionally, with the exception of the West which shows a downward trend in the high leverage domain, the average standardized residuals stay roughly centered on 0 across all leverage values.

Autocorrelation and Partial Autocorrelation. Autocorrelation and partial autocorrelation plots test for correlation between a time series with incrementally lagged versions of itself. Independence of observations can be inferred from insignificant autocorrelation in lags other than zero (as data will always be correlated with itself). The Northeast, Midwest, South and West all have decaying but significant autocorrelations up to lags of 4, 4, 3, and 3 respectively. For partial autocorrelation, this changes to 2, 1, 1, and 2, suggesting that dependence is highly localized to narrow shifts from the initial series. Low lag correlation, indicating dependence between consecutive death rates, is to be expected in our context as opioid death rates do not behave randomly - after all we know that the epidemic is characterized by increases in opioid mortality. All plots also show some significant lags beyond small shifts, but these are not consistently on timescales we would expect when working with monthly data (i.e. 6 or 12 months signifying a half or full year) with the exception of significant autocorrelation at the 12 month mark in the West, and significant autocorrelation and partial autocorrelation at 24 months in the Northeast and West. It is unclear as to what the underlying explanation for these sporadic significant lags is, but we believe that they are not overly concerning. The intervention time points chosen may also be factoring into the patterns observed.

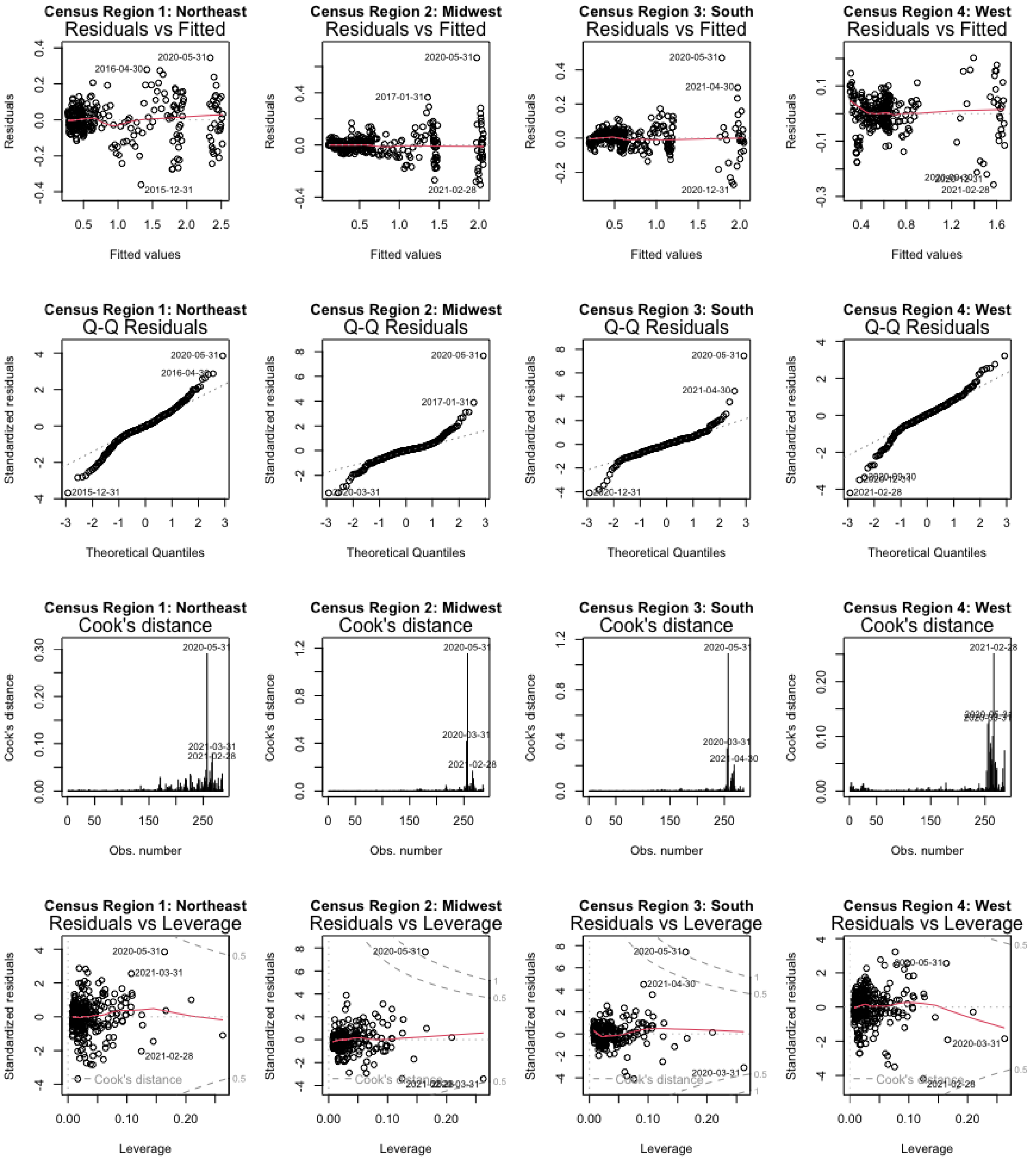

Fig S3-1. Standard linear regression diagnostics for the foundational ITS model.
Residual, normal Q-Q, Cook's distance, and leverage plots. Note the varying y-axis scales.

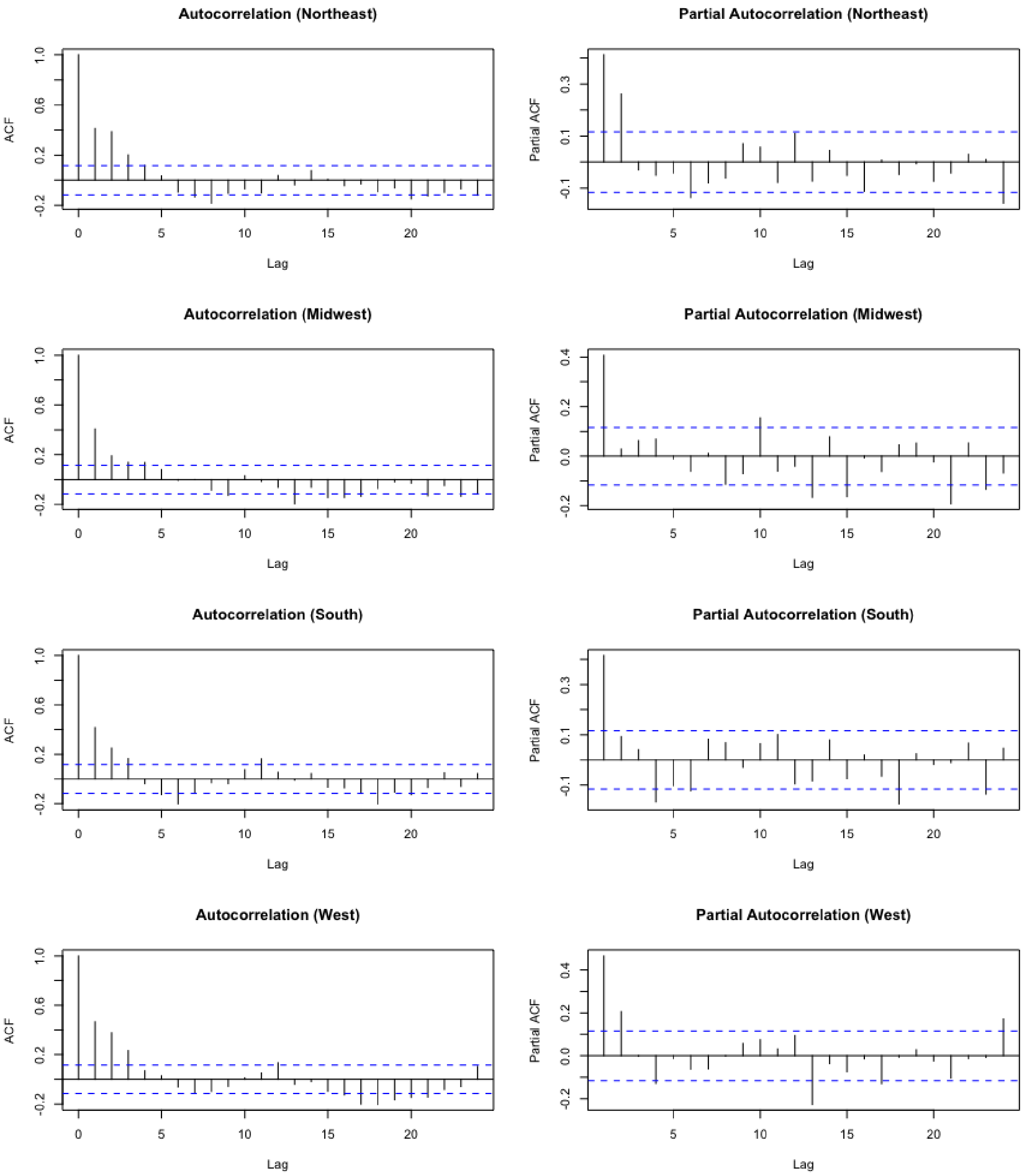

Fig S3-2. Autocorrelation and partial autocorrelation plots for the foundational ITS model. The blue dashed lines represent $\alpha = 0.05$ thresholds for significant autocorrelation function (ACF) and partial ACF values.

Gender-Stratified Model

We now work through diagnostics for an ITS model with linear regression to check whether the necessary assumptions are satisfied for the models in each region. Fig S3-3 shows standard plots addressing linearity, homoscedasticity, normality, and influential observations, with points coloured by gender. Fig S3-4 shows autocorrelation and partial autocorrelation.

Residuals. The residual plots for the Midwest, South, and West have an average residual line consistently aligned with $y = 0$, indicating that linearity is satisfied. The Northeast demonstrates an increasing pattern in residuals as the fitted values increase, which suggest that the model actually underestimates those death rates for the most recent stage of the opioid epidemic. Heteroscedasticity is most prominent in the Midwest and South, along with a wider range in residual values comparatively. Most of the heteroscedasticity can be attributed to the male data, which is expected due to higher variation in those death rates. The male opioid death rate from May 2020 produces the most drastic residual for the Northeast, Midwest, and South, exceeding 0.5 in those cases. Interestingly, the residuals of greatest magnitude in the West are all negative. Again, we note gaps in fitted values as a result of the immediate shifts in death rates during interventions.

Normal Q-Q. All four Q-Q plots suggest that the residuals belong to a heavy-tailed distribution, with better symmetry in the Northeast and South. With that said, the main culprits of asymmetry in the Midwest and South are a few outlier residuals as opposed to a systemic bias. The bulk of the datapoints fall healthily within the $(-2, 2)$ interval which is a good sign.

Cook's Distance. As expected, the data points with very high Cook's distances are all attributed to monthly opioid-related death rates from the male demographic. In particular, the male observations for March and May 2020 are in the top three Cook's distance values for all CRs. As the gender-stratified model now has 10 additional parameters due to the interaction term, the threshold value is $C_k = \frac{8}{286-2(20)} \approx 0.033$. All of the female Cook's distances appear to fall under this threshold, which is unsurprising as the opioid deaths are heavily skewed towards the male demographic. There are also larger jumps in opioid-related death rate amongst males due to COVID-19, a defining element of the model.

Leverage. Looking at the central tendencies, there are biases to positive residuals in the Northeast as the leverage values increase, which indicates that some of the higher leverage points may pull the model upwards, whilst the opposite pattern occurs in the West. The high influence and high leverage combination only occurs for the May 2020 male opioid-related death rates in the Midwest and South along the 1-threshold, though the corresponding point for March 2020 falls just under the 0.5-threshold. There are female opioid-related death rate observations with high leverage, but they are not combined with high influence.

Autocorrelation and Partial Autocorrelation. The autocorrelation and partial autocorrelation plots do not show any sign of significant seasonal patterns or other notable intervals. However, there is dependence between consecutive observations, which is unsurprising as there is a clear increasing pattern in opioid-related death rates over time.

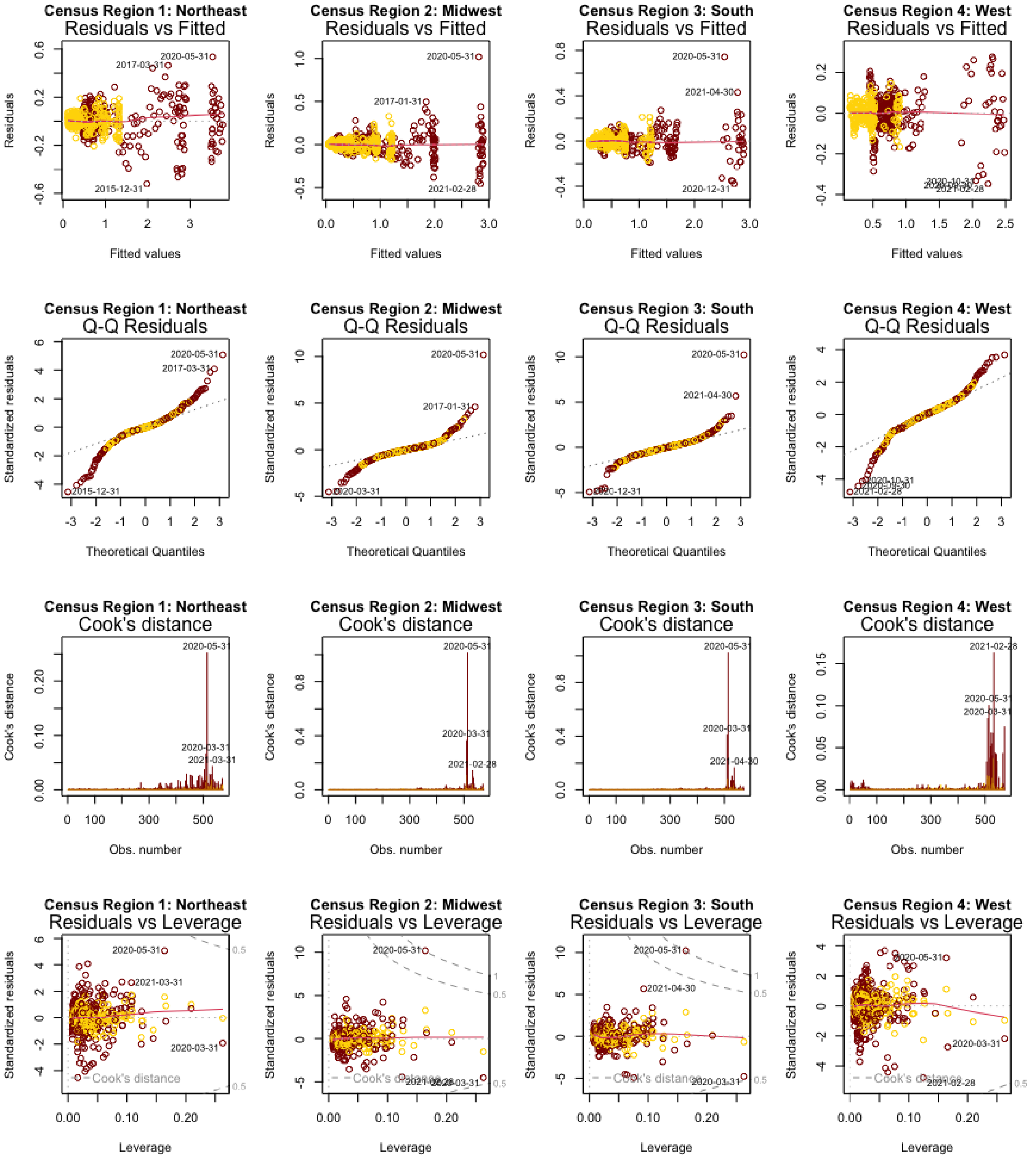

Fig S3-3. Standard linear regression diagnostics for the gender-stratified ITS model. Residual, normal Q-Q, Cook's distance, and leverage plots. Female datapoints are in yellow, and male datapoints are in dark red. Note the varying y-axis scales.

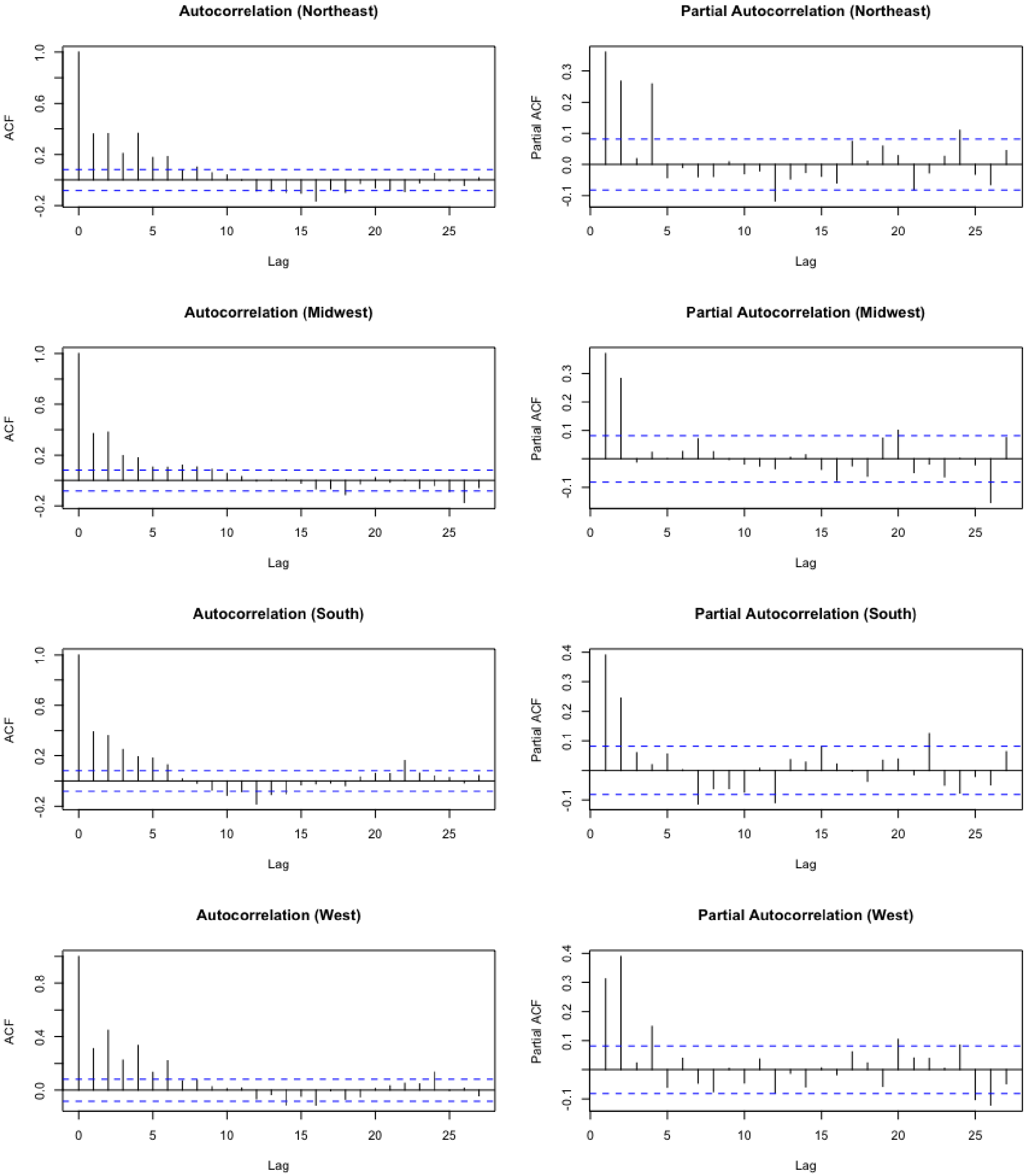

Fig S3-4. Autocorrelation and partial autocorrelation plots for the gender-stratified ITS model. The blue dashed lines represent $\alpha = 0.05$ thresholds for significant ACF and partial ACF values.
