# Supplementary material for: Evolution of an epidemic: Understanding the opioid epidemic in the United States and the impact of the COVID-19 pandemic on opioid-related mortality
Source: PLoS One. 2024 Jul 9;19(7):e0306395. doi: 10.1371/journal.pone.0306395 (PMC11233025; doi:10.1371/journal.pone.0306395)
Supplement: S4 Appendix — (PDF) [file pone.0306395.s004.pdf]

S4 Appendix

Sensitivity Analyses

We conduct sensitivity analyses in order to assess the robustness of our estimates for cumulative excess opioid-related deaths from March 2020 to October 2022. Our model uses ITS with linear regression, so we compare with Poisson regression using an equation analogous to the original ITS model with respect to the log of opioid-related death counts. Quasi-Poisson was omitted as it produces identical results to its Poisson counterpart; it adds a scale parameter, but this does not impact the line of best fit or the bootstrapped intervals. Additionally, the original analysis utilized data from January 1999 to October 2022, so we test our linear regression ITS model on two reduced datasets. The first dataset has a reduced temporal scope, and starts in January 2015 to evaluate sensitivity to historical data. The next dataset reduces the model input by eliminating the COVID-19 data, meaning our model is fit to the pre-COVID-19 era and extrapolates ahead. For both of the reduction datasets, irrelevant terms are dropped from the model (e.g. heroin terms are not needed for a dataset which starts in January 2015). The models are summarized in Table S4-1.

Table S4-1. Specifications of sensitivity analyses.

Model Name	Equation	Errors	Input Data
Our Model	$y_t = \beta_0 + \beta_1 t + \beta_2 \mathbb{I}_t^{(\text{Heroin})} + \beta_3 P_t^{(\text{Heroin})} + \beta_4 \mathbb{I}_t^{(\text{Fentanyl})} + \beta_5 P_t^{(\text{Fentanyl})} + \beta_6 P_t^{(\text{PHE})} + \beta_7 \mathbb{I}_t^{(\text{COVID})} + \beta_8 P_t^{(\text{COVID})} + \beta_9 P_t^{(\text{CHW})} + \epsilon_t$	Normal	January 1999 - October 2022
Poisson	$\log(d_t) = \beta_0 + \beta_1 t + \beta_2 \mathbb{I}_t^{(\text{Heroin})} + \beta_3 P_t^{(\text{Heroin})} + \beta_4 \mathbb{I}_t^{(\text{Fentanyl})} + \beta_5 P_t^{(\text{Fentanyl})} + \beta_6 P_t^{(\text{PHE})} + \beta_7 \mathbb{I}_t^{(\text{COVID})} + \beta_8 P_t^{(\text{COVID})} + \beta_9 P_t^{(\text{CHW})} + \epsilon_t$	Poisson	January 1999 - October 2022
Reduced Scope	$y_t = \beta_0 + \beta_1 t + \beta_6 P_t^{(\text{PHE})} + \beta_7 \mathbb{I}_t^{(\text{COVID})} + \beta_8 P_t^{(\text{COVID})} + \beta_9 P_t^{(\text{CHW})} + \epsilon_t$	Normal	January 2015 - October 2022
Reduced Input	$y_t = \beta_0 + \beta_1 t + \beta_2 \mathbb{I}_t^{(\text{Heroin})} + \beta_3 P_t^{(\text{Heroin})} + \beta_4 \mathbb{I}_t^{(\text{Fentanyl})} + \beta_5 P_t^{(\text{Fentanyl})} + \beta_6 P_t^{(\text{PHE})} + \epsilon_t$	Normal	January 1999 - February 2020

Cumulative excess opioid-related deaths estimates (denoted as “oCED”) with 95% bsCIs are shown graphically in Fig S4-1, broken down into all region and gender combinations for each model. The corresponding numerical results are contained in Table S4-2 and Table S4-3 for Poisson and Normal errors respectively. In these tables, we also present the percentage change (denoted as “%diff”) for the means of each benchmarking method relative to those from our model, calculated as

$$\%\text{diff} = \left(\frac{\text{oCED}_{\text{Comparison}} - \text{oCED}_{\text{Original}}}{\text{oCED}_{\text{Original}}} \right) \times 100.$$

A positive %diff value indicates that the alternate model yielded an overestimate of oCED compared to our model, and vice versa.

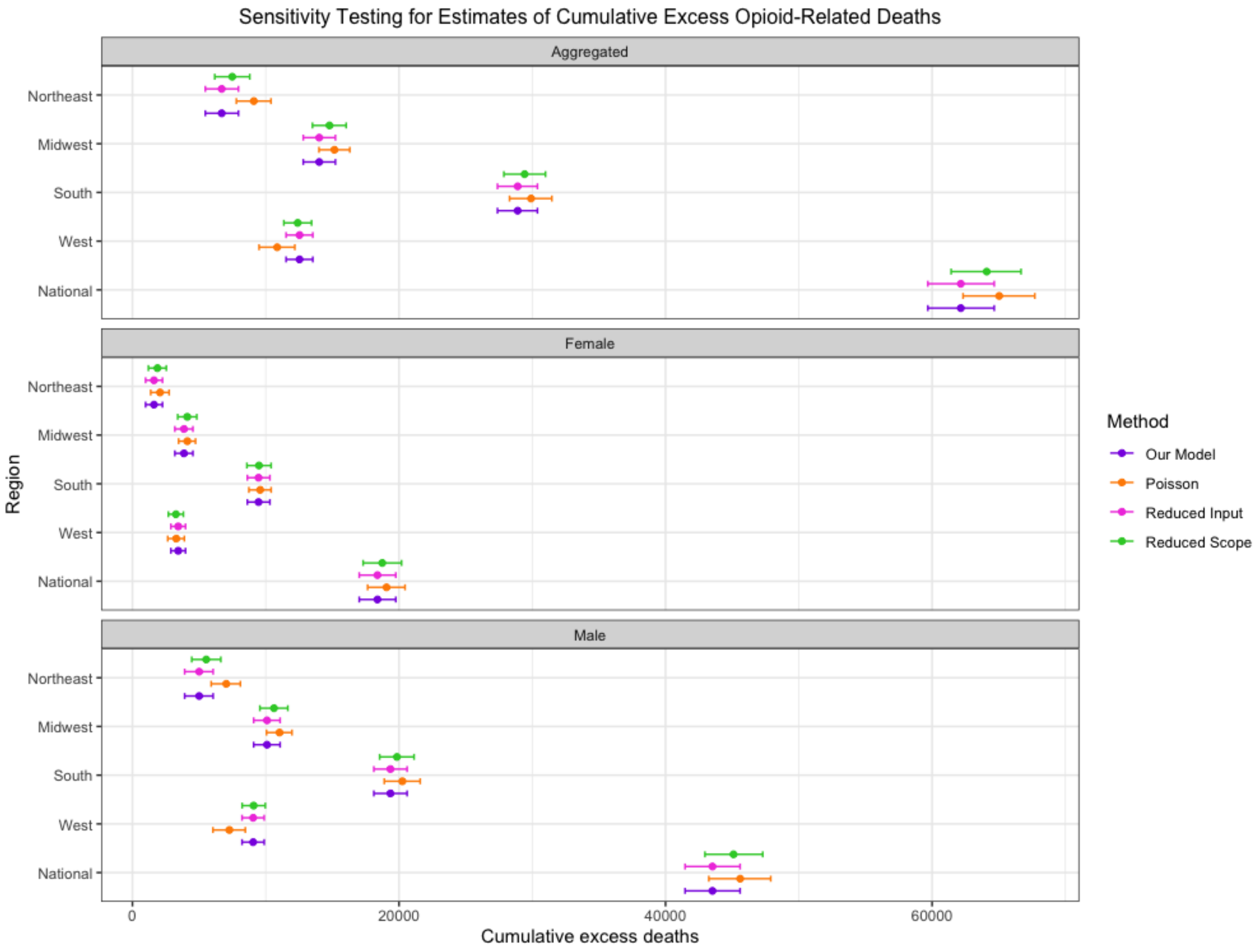

Fig S4-1. 95% bsCIs for estimates of cumulative excess opioid-related deaths under four models, March 2020 to October 2022.
Intervals are grouped by region and panelled by gender.

Table S4-2. Model comparisons for estimates of cumulative excess opioid-related deaths, March 2020 to October 2022.

Group		Our Model	Poisson	
Region	Gender	oCED	oCED	%diff
Northeast	–*	6,699 (5,468–7,954)	9,107 (7,799–10,398)	36.0%
Midwest	–	14,015 (12,817–15,231)	15,155 (13,998–16,310)	8.1%
South	–	28,904 (27,390–30,390)	29,910 (28,295–31,470)	3.5%
West	–	12,538 (11,529–13,536)	10,861 (9,502–12,185)	-13.4%
National	–	62,156 (59,679–64,662)	65,034 (62,323–67,707)	4,6%
Northeast	Female	1,617 (987–2,254)	2,073 (1,373–2,753)	28.2%
Midwest	Female	3,869 (3,182–4,540)	4,119 (3,464–4,734)	6.5%
South	Female	9,463 (8,621–10,314)	9,585 (8,738–10,408)	1.3%
West	Female	3,434 (2,883–3,984)	3,288 (2,649–3,897)	-4.2%
National	Female	18,382 (17,017–19,761)	19,066 (17,647–20,454)	3.7%
Northeast	Male	5,006 (3,919–6,056)	7,037 (5,909–8,102)	40.6%
Midwest	Male	10,098 (9,095–11,080)	11,036 (10,062–11,971)	9.3%
South	Male	19,358 (18,114–20,609)	20,254 (18,896–21,591)	4.6%
West	Male	9,057 (8,223–9,885)	7,273 (6,039–8,470)	-19.7%
National	Male	43,520 (41,467–45,594)	45,600 (43,249–47,895)	4.8%

* Groups with a "–" in the Gender column signify results from the aggregate model as opposed to the gender-stratified model.

Table S4-3. Input data-scope comparisons for estimates of cumulative excess opioid-related deaths, March 2020 to October 2022.

Group		Our Model	Reduced Input		Reduced Scope	
Region	Gender	oCED	oCED	%diff	oCED	%diff
Northeast	–*	6,699 (5,468–7,954)	6,699 (5468–7954)	0.0%	7,488 (6,176–8,803)	11.8%
Midwest	–	14,015 (12,817–15,231)	14,015 (12,817–15,231)	0.0%	14,785 (13,508–16,039)	5.5%
South	–	28,904 (27,390–30,390)	28,904 (27,390–30,390)	0.0%	29,424 (27,872–30,993)	1.8%
West	–	12,538 (11,529–13,536)	12,538 (11,529–13,536)	0.0%	12,401 (11,362–13,441)	-1.1%
National	–	62,156 (59,679–64,662)	62,156 (59,679–64,662)	0.0%	64,097 (61,428–66,674)	3.1%
Northeast	Female	1,617 (987–2,254)	1,617 (987–2,254)	0.0%	1,877 (1,199–2,544)	16.1%
Midwest	Female	3,869 (3,182–4,540)	3,869 (3,182–4,540)	0.0%	4,114 (3,396–4,829)	6.3%
South	Female	9,463 (8,621–10,314)	9,463 (8,621–10,314)	0.0%	9,493 (8,589–10,406)	0.3%
West	Female	3,434 (2,883–3,984)	3,434 (2,883–3,984)	0.0%	3,262 (2,696–3,824)	-5.0%
National	Female	18,382 (17,017–19,761)	18,382 (17,017–19,761)	0.0%	17,846 (17,308–20,192)	2.0%
Northeast	Male	5,006 (3,919–6,056)	5,006 (3,919–6,056)	0.0%	5,536 (4,450–6,632)	10.6%
Midwest	Male	10,098 (9,095–11,080)	10,098 (9,095–11,080)	0.0%	10,623 (9,563–11,659)	5.2%
South	Male	19,358 (18,114–20,609)	19,358 (18,114–20,609)	0.0%	19,848 (18,548–21,131)	2.5%
West	Male	9,057 (8,223–9,885)	9,057 (8,223–9,885)	0.0%	9,092 (8,231–9,972)	0.4%
National	Male	43,520 (41,467–45,594)	43,520 (41,467–45,594)	0.0%	45,099 (42,957–47,294)	3.6%

* Groups with a "–" in the Gender column signify results from the aggregate model as opposed to the gender-stratified model.

Results

Based on the vast majority of positive %diff values, our model tends to yield more conservative estimates of cumulative excess opioid-related deaths. This is advantageous because, even in a conservative regime, there is clear quantitative evidence that the opioid epidemic has worsened under COVID-19. In reality, the knock-on effects of COVID-19 on the opioid epidemic are likely more severe than our predictions showed, further strengthening the need for public health interventions.

There is overlap between all of the 95% bootstrapped confidence intervals in Fig S4-1, indicating that our model is largely consistent with Poisson regression and a similar analyses with reduced data. The largest offsets for the benchmarking models occur in the Northeastern estimates due to smaller death counts and thus more volatility in %diff. The Poisson model underestimates cumulative excess opioid-related deaths for the male and aggregated groups in the West compared to our model, and thus could be used as a lower bound for interpreting results.

The identical results between our model and the reduced input model are expected as the counterfactual computations eliminate the model terms associated with COVID and the CHW; therefore COVID-19 and policy responses to COVID-19 are not included in the prediction model for excess mortality. Because of the way the ITS equation is set up mathematically, dropping out those latter terms results in a continuation of the pre-Covid trends. As such, excess mortality compares observed deaths to the pre-Covid trends extrapolated beyond March 2020, and there is no change in cumulative excess deaths or the corresponding bsCIs.

Overall, the sensitivity analyses corroborate our original analysis, and strengthen the applicability of our results towards informing public health and policy regarding the opioid epidemic.
